# Equilibrium Thermodynamics of Macropa Complexes with Selected Metal Isotopes of Radiopharmaceutical Interest

**DOI:** 10.1021/acs.inorgchem.3c01983

**Published:** 2023-09-13

**Authors:** Magdalena K. Blei, Lukas Waurick, Falco Reissig, Klaus Kopka, Thorsten Stumpf, Björn Drobot, Jerome Kretzschmar, Constantin Mamat

**Affiliations:** ‡ Helmholtz-Zentrum Dresden-Rossendorf, Institute of Radiopharmaceutical Cancer Research, Bautzner Landstraße 400, D-01328 Dresden, Germany; § TU Dresden, Faculty of Chemistry and Food Chemistry, D-01062 Dresden, Germany; ⊥ Helmholtz-Zentrum Dresden-Rossendorf, Institute of Resource Ecology, Bautzner Landstraße 400, D-01328 Dresden, Germany; ∥ National Center for Tumor Diseases, University Cancer Center, University Hospital Carl Gustav Carus Dresden, Fetscherstraße 74, D-01307 Dresden, Germany; ¶ German Cancer Consortium, Partner Site Dresden, Fetscherstraße 74, D-01307 Dresden, Germany

## Abstract

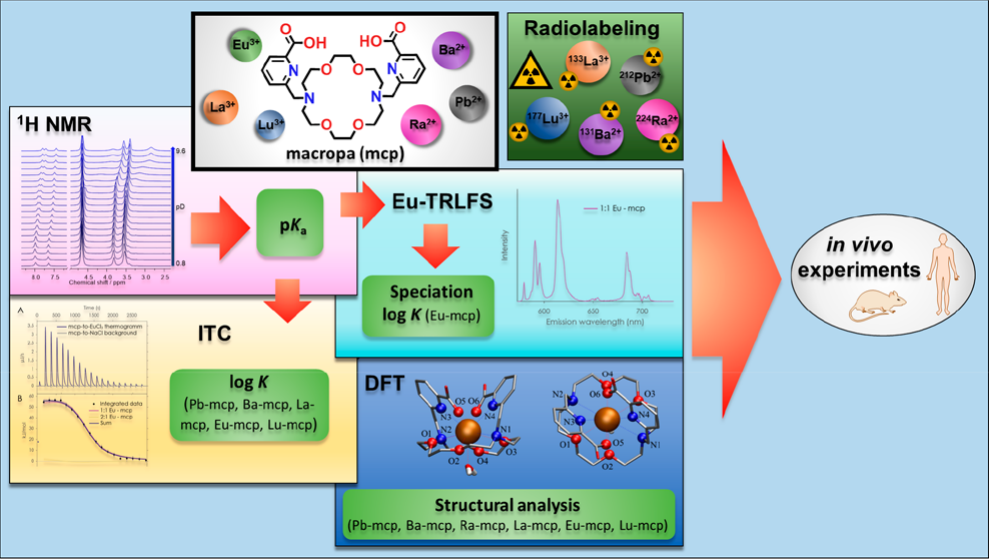

To pursue the design of *in vivo* stable chelating systems for radiometals, a concise and straightforward method toolbox was developed combining NMR, isothermal titration calorimetry (ITC), and europium time-resolved laser-induced fluorescence spectroscopy (Eu-TRLFS). For this purpose, the macropa chelator was chosen, and Lu^3+^, La^3+^, Pb^2+^, Ra^2+^, and Ba^2+^ were chosen as radiopharmaceutically relevant metal ions. They differ in charge (2+ and 3+) and coordination properties (main group vs lanthanides). ^1^H NMR was used to determine four p*K*_a_ values (±0.15; carboxylate functions, 2.40 and 3.13; amino functions, 6.80 and 7.73). Eu-TRLFS was used to validate the exclusive existence of the 1:1 M^*n*+^/ligand complex in the chosen pH range at tracer level concentrations. ITC measurements were accomplished to determine the resulting stability constants of the desired complexes, with log *K* values ranging from 18.5 for the Pb-mcp complex to 7.3 for the Lu-mcp complex. Density-functional-theory-calculated structures nicely mirror the complexes’ order of stabilities by bonding features. Radiolabeling with macropa using ligand concentrations from 10^–3^ to 10^–6^ M was accomplished by pointing out the complex formation and stability (^212^Pb > ^133^La > ^131^Ba ≈ ^224^Ra > ^177^Lu) by means of normal-phase thin-layer chromatography analyses.

## Abstract 2

Complexes formed by the chelator macropa and the metal ions of La, Eu, Lu, Pb, and Ba were analyzed by NMR, isothermal titration calorimetry (ITC), europium time-resolved laser-induced fluorescence spectroscopy (Eu-TRLFS), and density functional theory to determine the p*K*_a_ values for macropa (2.40, 3.13, 6.80, and 7.73) and the stability constants (log *K*) of the respective complexes (Pb-mcp, 18.5; La-mcp, 13.9; Eu-mcp, 13.0; Ba-mcp, 11.0; Lu-mcp, 7.3). Additionally, structural information was obtained, and radiolabeling of macropa with selected radionuclides was realized with a complex stability gradation in the order of ^212^Pb > ^133^La > ^131^Ba ≈ ^224^Ra > ^177^Lu.

## 1 Introduction

Radiometals and especially the design of chelating systems to create stable radiometal complexes continue to be an important element of radiopharmaceutical development for both diagnostic and therapeutic applications in nuclear medicine.^1,2^ Since the development of technetium-99m as a still-dominating diagnostic radionuclide and the emergence of the ^99^Mo/^99m^Tc generator in the 1960s,^3^ several radiometal-based nuclides have been identified and implemented as potential radiopharmaceuticals.^4^ A critical aspect of radiometal-based radiopharmaceuticals is their stability under *in vivo* conditions. The radiometal-coordinating ligand is the key to determining the radiometal complex stability. Release of the radiometal ion should be avoided to prevent the accumulation of free radiometal *in vivo* (off-target accumulation).^5^ So far, no single chelator is known to be ideal for all metals or radioconjugates. Hence, discovering appropriate complexation systems is of unabated interest. The precise determination of the thermodynamic association constants (log *K*) allows the prediction of *in vivo* stability of radiometal complexes at the radiotracer level. In addition, thermodynamic studies can help to identify conditions under which the desired thermodynamic product dominates over a possibly less stable kinetic product. It should be noted that the kinetic stability of the complex formed cannot be directly determined from complex formation constants because the complex formation constant corresponds to the ratio of the association and dissociation rates.

The stability of the radiometal complex *in vivo* is not just influenced by the chelator.^6^ Moreover, the biodistribution and clearance are affected as well. In this regard, the net charge and hydrophilicity of the resultant radiometal conjugate have an impact, which has been shown for Cu^2+^ complexes conjugated to the same targeting moiety.^7−10^ It is therefore important to search for appropriate chelators to match both the radiometal and biological target in order to obtain the best target-to-nontarget uptake ratio of the radiopharmaceutical for either imaging or therapy. Mostly, encapsulation by the multidentate and macrocyclic chelator 2,2′,2″,2‴-(1,4,7,10-tetraazacyclododecane-1,4,7,10-tetrayl)tetraacetic acid (DOTA) as the most used standard ligand is required.^11^ It works well for radiometals, namely, ^43/44/47^Sc, ^177^Lu, ^111^In, and ^67/68^Ga, but it is not the best choice for other radiolanthanides like ^133^La or radioactinides like ^225^Ac and even worse for radionuclides from main group 2 metals such as ^90^Sr, ^131^Ba,^12^ and ^223/224^Ra.^13^

**Figure 1:**
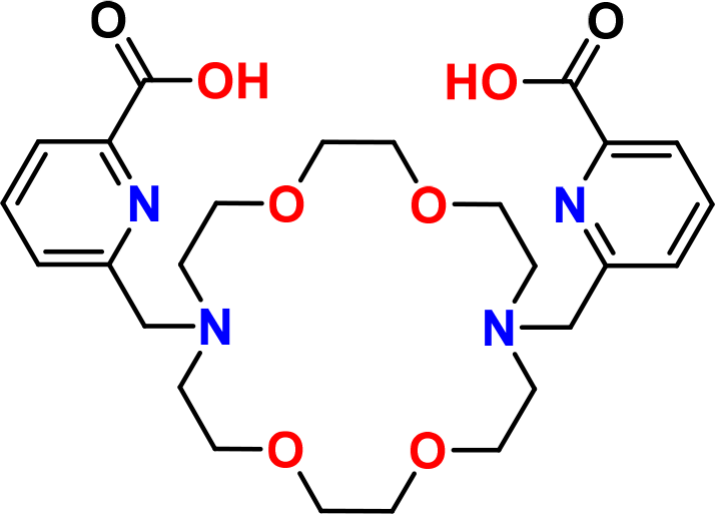
Molecular structure of the macropa chelator.

Macropa (mcp) as a macrocyclic chelator, originally known as H2bp18c6 and invented for actinide and lanthanide separation,^14,15^ with two pendant picolinic side arms (Figure 1) has entered the field of radiopharmaceutical sciences especially for the complexation of ^225^Ac,^16−18^^133^La,^19^^213^Bi,^20,21^^131^Ba,^22^ and ^223/224^Ra.^23^ Macropa’s superiority over DOTA in forming highly stable complexes with the trivalent cation of ^225^Ac has been shown.^24^ Moreover, water-soluble complexes with divalent cations of barium^25^ and radium^23^ in conjunction with macropa are known. Due to the lack of stability data for macropa-derived complexes, especially for radiopharmaceutical *in vivo* applications, extensive research on new chelating systems, combined with precise analytical methods for the log *K* determination, is still necessary.

Various attempts were made in the past to determine the complex stability of the (radio)metal complexes. Different analytical methods are known to generally measure the association constants such as UV/vis titration, potentiometry, or NMR titrations.^11^ However, these methods are sometimes carried out at their limit of validation.^26−28^ The often low concentration of either the (radio)metal or the ligand falls below the limit of detection for these methods. Other standard methods like UV/vis or NMR are not appropriate for Ra^2+^ because a high amount of metal salt is required. Additionally, radiochemical two-phase extractions are not suitable due to the high solubility of the complexes in aqueous media.

To overcome these obstacles, our aim was to establish a reliable method toolbox consisting of a combination of NMR, isothermal titration calorimetry (ITC), and europium time-resolved laser-induced fluorescence spectroscopy (Eu-TRLFS) for the accurate determination of association constants for complexes formed with macropa and selected cations. The latter thereby comprise Pb^2+^ and Ba^2+^ as well as the (radio)lanthanides La^3+^, Eu^3+^, and Lu^3+^, which are commonly applied as radiometal ions for diagnostic and therapeutic purposes in radiopharmaceutical applications (except of Eu^3+^). Moreover, measurements with concentrations in the micromolar range are possible, enabling a more realistic prediction of the radiopharmaceutical’s behavior at tracer-level concentrations.

## 2 Experimental Section

### 2.1 Synthesis and Preparation of Solutions

All chemicals were used without further purification. The synthesis of the macropa ligand was accomplished according to published methods.^14,29^ NMR samples were prepared with deuterated solvents (Deutero) D_2_O (99.95% D) as well as D_2_O solutions of both DCl (38% in D_2_O with 99% D) and NaOD (40% in D_2_O with 99% D) to adjust the pD using a pH meter (VWR pHenomenal MU 6100 L) equipped with a pH electrode (WTW SenTix Mic) and corrected for deuterium according to the common relationship pD = pH meter reading + 0.4.^30^ A stock solution of 2 mM macropa and 0.2 M NaCl was prepared by weighing and dissolving the required amounts in D_2_O. Samples were prepared by taking 1 mL of the stock solution and adjusting the pD and total volume to 2 mL to finally yield samples of 1 mM macropa and 0.1 M NaCl, with a pD range from 0.8 to 9.6.

The samples for TRLFS and ITC were prepared at 25 °C with an ionic strength of NaCl set to 0.1 M. For all samples, the pH was adjusted using NaOH and HCl and a pH meter (VWR pHenomenal MU 6100 L) equipped with a pH electrode (WTW SenTix Mic).

### 2.2 NMR Spectroscopy

All NMR spectra were recorded at 25 °C using Agilent DD2-600 and 400MR DD2 systems, operating at 14.1 and 9.4 T, with corresponding resonance frequencies of 599.8 and 399.8 MHz for ^1^H and 150.8 and 100.6 MHz for ^13^C, respectively, using 5 mm NMR probes. ^1^H NMR spectra were measured by accumulating 16–128 scans, depending on the concentrations and line widths, using 2 s of acquisition time and a relaxation delay, respectively, applying a 2 s presaturation pulse on the water (HDO) resonance for water signal suppression. NMR spectra were processed with *MestReNova*, version 14.2.3 (Mestrelab Research SL).^31^ The creation of graphs for numerical data visualization and data fitting by nonlinear sigmoidal dose–response fit algorithm were performed with *Origin 2020*, version 9.7.0.185 (OriginLab Corp.). Furthermore, macropa complexes of La^3+^, Eu^3+^, Lu^3+^, Pb^2+^, and Ba^2+^ were prepared.

### 2.3 TRLFS

For TRLFS, a pulsed Nd:YAG-OPO laser system (Ekspla, NT230, 50 Hz, ∼5 ns pulse, 1.1 mJ/pulse) with an excitation wavelength of 394 nm was used. For the detection of Eu^3+^ luminescence, an Andor iStar ICCD camera (Lot-Oriel Group) connected to the rear end of a spectrograph (Oriel MS 257 monochromator, a 300 lines/mm grid, a gate width of 300 μs) was used. The luminescence decay was detected by measuring 21 different temporal offsets to the laser beam using a linearly increasing step size [7 + 7*x* (μs), 0–1610 μs] and an initial delay of 12 μs to suppress emission from higher states (^5^D_1_). Five solutions with 10 μM EuCl_3_ and 10 μM macropa in a 0.1 M NaCl solution in the pH range from 1.7 to 11.6 were prepared. Furthermore, for titration, a 500 μM macropa with 10 μM EuCl_3_ (0.1 M NaCl) solution and a 10 μM EuCl_3_ (0.1 M NaCl) solution at pH 3.5 were prepared.

In addition to the measurement at ambient temperatures, samples (10 μM EuCl_3_, 25 μM macropa, *I* = 0.1 M NaCl, pH 5) were measured under cryogenic conditions (<40 K, a closed-cycle helium refrigerated cryostat). The above-described laser system was used for excitation. Emission detection was performed with different grids (300, 600, and 1200 lines/mm). Due to longer lifetimes, the linear increasing step size was set to 15 + 15*x* (μs) [0–3450 μs].

All TRLFS data sets were analyzed by parallel factor analysis (PARAFAC) using an implemented N-way toolbox^32^ for MATLAB with modifications as previously described.^33,34^ The luminescence decays of individual species were constrained to be exponential, and the species distribution had to reflect a speciation of the Eu-macropa system. The p*K*_a_ values of macropa, which were determined by ^1^H NMR spectroscopy, were considered for the speciation calculation to extract complex stability constants log *K*.

### 2.4 Isothermal Titration Calorimetry (ITC)

MicroCal PEAQ-ITC (Malvern Panalytical) with a cell volume of approximately 200 μL was used for calorimetry. The titrant was injected into the sample cell by an automated syringe (40 μL) at 25 °C under continuous stirring (750 rpm) and was titrated in 0.5–2 μL steps in 19 aliquots with a time interval of 150 s into the sample cell. The syringe was filled with 500 μM macropa in a 0.1 M NaCl solution, and the sample cell contained 50 μM metal in a 0.1 M NaCl solution at the same specific pH (±0.1). An analogous setup without metal was used as the background measurement.

ITC data where analyzed using a MATLAB code based on speciation calculations^35^ and equations (e.g., displaced volume) provided by Malvern. Unless otherwise stated, the data were evaluated globally. The estimation of errors and the goodness of the model was performed with a Monte Carlo approach as previously described.^36^

### 2.5 Radiolabeling

***Caution!**^212^Pb, ^133^La, ^131^Ba, ^224^Ra, and ^177^Lu, and their radioactive decay products, represent α-, β-, and γ-emitting radionuclides. Special attention should be paid when working with unsealed radionuclides to avoid unnecessary contamination and incorporation. Only persons who are adequately trained or experienced are allowed to work with radioactive material in laboratories that are authorized to use radioactive material. Hence, all studies with these radionuclides were conducted in laboratories equipped with continuous air monitors, fume hoods (certified), and monitoring equipment appropriate for α-, β-, and γ-radiation detection. Entrance to the laboratory space (controlled area) was controlled with a hand and foot monitoring instrument for α-, β-, and γ-emitting isotopes and a personnel contamination monitoring station.*

The production of ^131^Ba and ^133^La was carried out at the TR-FLEX (ACSI) cyclotron at HZDR and is described elsewhere.^22,37^^177^Lu was commercially supplied by ITM as a [^177^Lu]LuCl_3_ solution. ^224^Ra and ^212^Pb were obtained from the ^228^Th/^224^Ra generator, as described elsewhere.^38,39^ Radiolabeling was performed using 100 kBq of ^131^Ba, ^133^La, ^224^Ra, ^212^Pb, or ^177^Lu. The respective ligand stock solution (10^–2^–10^–4^ M) in 0.2 M NH_4_OAc buffer (pH 6) was used to reach the required ligand concentration (10^–3^–10^–6^ M). For comparability, all reaction mixtures were maintained at 25 °C for 1 h in a thermomixer at 600 rpm. Once the labeling reaction was finished, samples were taken out and analyzed via radio-TLC [thin-layer chromatography; two systems: normal phase –50 mM ethylenediaminetetraacetic acid (EDTA; pH 7) on silica plates and normal phase (cyano-modified)–70:30 acetonitrile/water on Alugram-CN plates].^17^ Radio-TLC plates were imaged by radiation-sensitive imaging plates and scanned by an Amersham Typhoon 5 (GE Healthcare).

### 2.6 Computational Calculations

Geometry optimizations using density functional theory (DFT) were performed in *Orca 5.0.3* to investigate the 1:1 complex of Ba^2+^, La^3+^, Eu^3+^, Lu^3+^, Pb^2+^, and Ra^2+^ with the macropa ligand, accompanied by a single water molecule within the coordination sphere.^40^ The initial geometries were extracted from crystal structures exhibiting 10 coordinative bonds between the central atom and the macropa ligand.^14,25,41^ Implicit solvation with water was incorporated using the conductor-like polarizable continuum model.^42^ The PBE0 hybrid functional was employed, along with two distinct basis sets.^43,44^ For the alkaline-earth metals Ba^2+^ and Ra^2+^, the aug-cc-pvTZ-DK3 all-electron basis set was utilized in conjunction with the DKH-def2-TZVPP basis set for all atoms except the metal centers.^45^ On the other hand, the def2-TZVPP basis set with effective core potentials for the central atoms was employed for optimizations involving La^3+^, Eu^3+^, Lu^3+^, and Pb^2+^.^46^ In contrast to the total energy of certain complex structures, the geometry information is comparable for calculations with different basis sets. Therefore, structural and visual analyses were performed after the DFT optimizations with *VMD 1.9.3*.^47^ It is noteworthy that using the same basis set for all metal complexes consistently led to convergence difficulties in the self-consistent-field calculations. Subsequent to the geometry optimization, a numerical vibrational frequency analysis was conducted for the Ba^2+^ and Ra^2+^ complexes, while an analytical approach was employed for the La^3+^, Eu^3+^, Lu^3+^, and Pb^2+^ complexes. This analysis ensured the absence of imaginary frequencies, thereby confirming the attainment of a local energy minimum.

## 3 Results and Discussion

Spectroscopic techniques, which are excellently applicable for studying (aqueous) complex formation from both the organic ligand and (luminescent) metal-ion perspectives, are combined with calorimetry to benefit from each method’s advantages to finally identify the real species along with their molecular structures and to obtain the corresponding reliable thermodynamic data.^48^ A prerequisite to obtaining robust thermodynamic constants is the determination of reliable p*K*_a_ values of the ligand. For this purpose, ^1^H NMR spectroscopy was used. Advantageously, structure-related information is provided by covering a wider pH range. For example, the determination of a p*K*_a_ value of about 1 is hardly feasible in potentiometric or calorimetric titrations because the detectability of the abstracted proton is poor against background H^+^ concentrations ranging between 0.1 and 1 M corresponding to pH values between 1 and 0, respectively. Also, NMR’s structure sensitivity allows for an assignment of abstracted H^+^ to the corresponding protolytic site. Additionally, the pH-dependent behavior of the ligand–Eu^3+^ system was investigated by TRLFS in order to verify the exclusive existence of one single pH-independent 1:1 mcp/metal complex. The suitable pH value for the concentration titrations to determine log *K* was established. The latter was then determined complementarily using ITC and TRLFS. The strength of these two methods is the extremely high sensitivity enabling experiments with analyte concentrations in the micromolar concentration range.

### 3.1 p*K*_a_ Determination by NMR Spectroscopy

To ascertain the protonation–deprotonation regime of macropa as a chelating system, p*K*_a_ values of the functional groups were determined by ^1^H NMR spectroscopy. This allows a good assessment of a pH range for the later TRLFS and ITC measurements. For the p*K*_a_ determination, samples with 1 mM macropa in a 0.1 M NaCl D_2_O solution covering the pD range from 0.8 to 9.6 were prepared. p*K*_a_ values of the ligand were determined according to procedures successfully applied to other ligand systems.^48−50^

**Figure 2:**
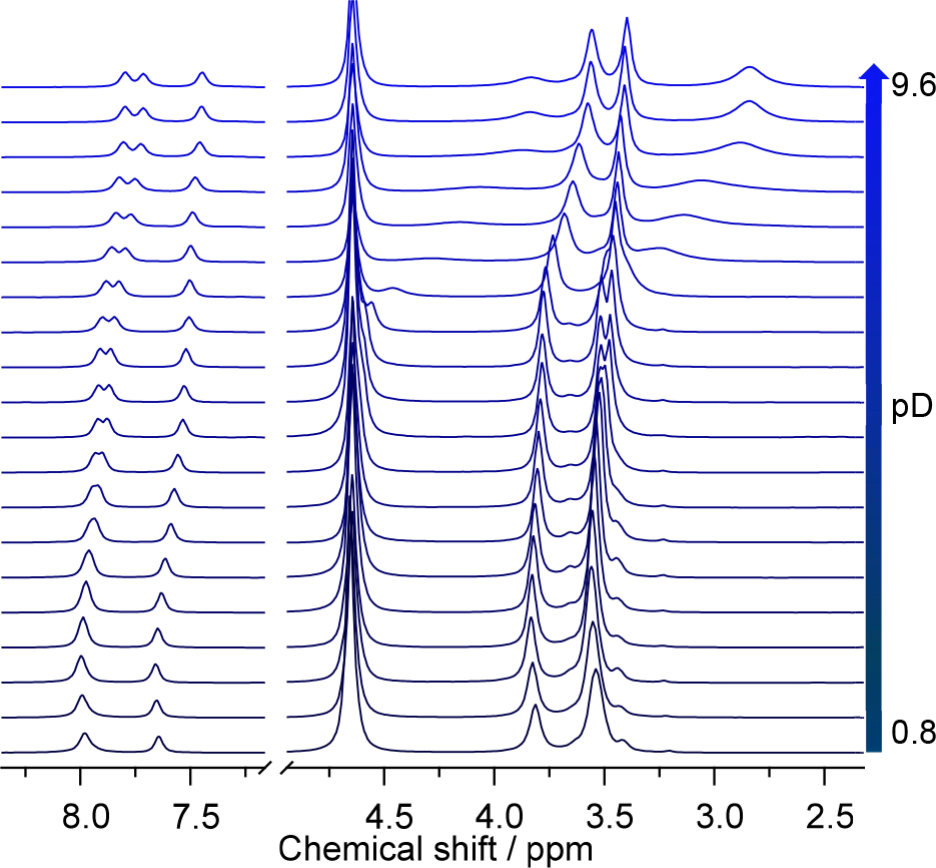
^1^H NMR pD-titration series of the macropa ligand obtained from 1 mM ligand in 0.1 M NaCl aqueous D_2_O solutions at (25 ± 1) °C in the pD range of 0.8–9.6 (from bottom to top). For clarity, only selected spectra and spectral regions of interest are shown. For better visualization of the broad signals, an exponential line broadening factor of 20 Hz was applied. The signal at 4.65 ppm is the residual HDO resonance, partly obscuring the benzylic methylene ^1^H signal.

Upon increasing pH, a successive ligand deprotonation takes place, resulting in a general upfield shift of the signals due to the higher electron density remaining with the ligand caused by the increasing anionic charge (Figure 2).

**Figure 3:**
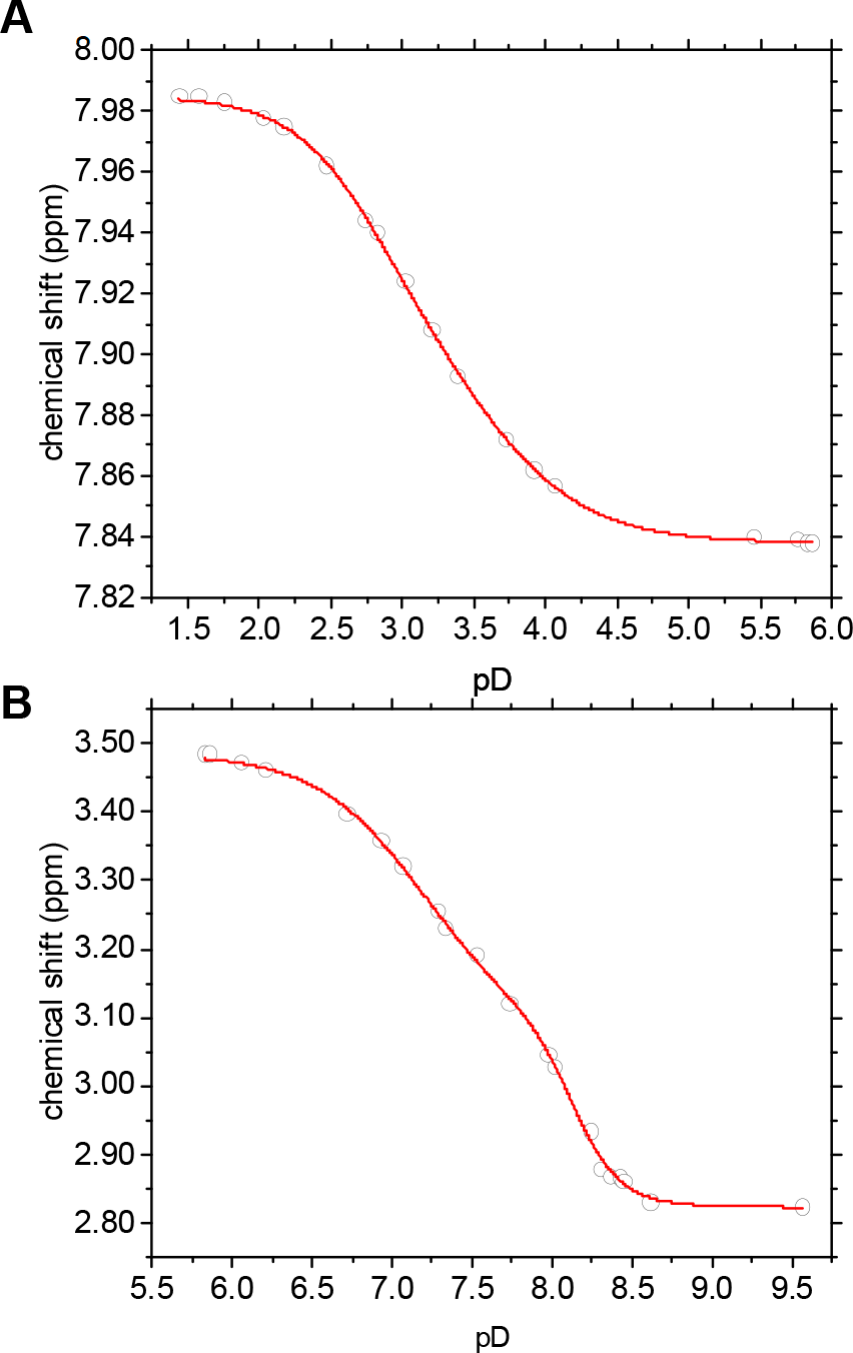
Graphs of pD-dependent ^1^H NMR chemical shift values (grey circles) based on the spectra shown in Figure 2, along with sigmoidal bidose–response fits (red lines) for p*K*_a_ determination. Signals associated with the aromatic residue (A) and macrocycle (B) are used as representative molecular probes to monitor the abstraction of protons from the carboxyl groups and amine nitrogen atoms, respectively.

The variable line widths (signal width at half-amplitude) are strong indicators of kinetic processes associated with protonation/deprotonation reactions. Especially, the signals of ^1^H nuclei in the direct vicinity reveal significant broadening at the pH range close to the amines’ p*K*_a_ values. The four inflections observed for ligand titration, in agreement with Roca-Sabio et al.,^14^ refer to two protons abstracted from the carboxylic groups in acidic media as well as two protons abstracted from the macrocyclic ring amine nitrogen atoms (ammonium form) under nearly neutral-to-alkaline conditions. ^1^H NMR titration data were evaluated in the corresponding δ_H_ versus pD plots by means of sigmoidal (bi)dose–response fit functions, as shown in Figure 3, with the respective inflection points representing p*K*_a_ values.

In order to correct the deuterium isotope effect in the p*K*_a_ value determination, a constant value of 0.4 was subtracted to obtain the p*K*_a_ values comparable to water solutions, according to the linear term in the pD determination from a pH meter reading.^51^ Determined p*K*_a_ values are summarized in Table 1.

**Table 1:** Determined Deuterium-Corrected p*K*_a_ Values of Macropa Obtained from NMR Spectroscopy at an Ionic Strength of *I* = 0.1 M NaCl in Comparison to the Literature Data

	**1**	**2**	**3**	**4**
	H_4_L^2+^ → H_3_L^+^	H_3_L^+^ → H_2_L^0^	H_2_L^0^ → HL^–^	HL^–^ → L^2–^
p*D*_a_ (D_2_O)	2.80	3.53	7.20	8.13
**p*K***_**a**_^a^	**2.40**	**3.13**	**6.80**	**7.73**
**p*K***_**a**_**(lit.)**^b^^,^(14)	**2.36**	**3.32**	**6.85**	**7.41**

a Estimated error values of ±0.15, considering the uncertainties arising from the pH electrode including temperature effects during calibration and measurement.

b Potentiometric titration (*I* = 0.1 M KCl).

### 3.2 Investigations of the Eu-mcp Complex by TRLFS

Thermodynamic investigations of metal complexes with high-affinity ligands reveal intrinsic difficulties. The affinity of the completely deprotonated ligands toward the metal ion of interest is in the range for which a useful concentration regime is below the detection limits of most spectroscopic techniques for direct measurement of the complex formation constants. The resulting species distributions consist of a linear increase in the complex concentration followed by a kink and a plateau (Figure S5). This fast saturation is not suitable for the direct determination of the complex formation constants because it does not allow for correct measurement of the equilibrium free metal concentration required for the law of mass action. Our approach is to perform the experiment at a lower pH, reducing the ligand’s affinity because of competition between the metal ion and protons for the functional groups. In that case, a direct accessibility of the log *K* values of these high-affinity ligand complexes is possible. Thus, it must first be validated that the complex under investigation remains stable in the considered pH range (Figure S4). The experiments were repeated at pH 3.5 with a reduced affinity of the ligand, leading to a smooth asymptotic saturation (Figure S6). By utilizing the exact p*K*_a_ values (Table 1), it is now possible to determine the log *K* values in a suitable concentration range, as previously demonstrated for nitrilotriacetic acid, EDTA, and ethylene glycol tetraacetic acid.^48^

### 3.3 Thermodynamic Investigations by TRLFS and ITC

**Figure 4:**
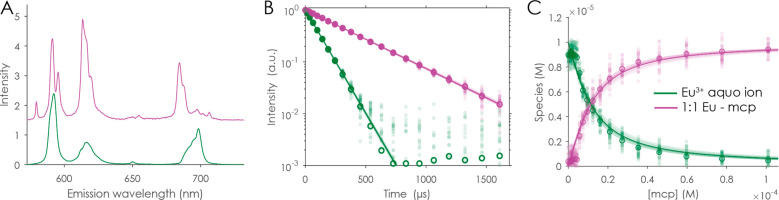
PARAFAC results of mcp titration (0–112 μM) to 10 μM EuCl_3_ in 100 mM NaCl at pH 3.5. Besides the Eu^3+^ aquo ion (green), the Eu-mcp complex (magenta) is clearly identified. Because of PARAFAC’s trilinearity, the emission spectra (A), luminescence decay of the Eu^3+^ aquo ion (109 ± 1.2 μs), 1:1 Eu-mcp complex (385 ± 4 μs) (B), and speciation (C) were simultaneously determined. The shaded data points were artificially created to be used in a Monte Carlo approach for the error estimation of the underlying model.

The combination of the discussed TRLFS titration of macropa to the Eu^3+^ solution at pH 3.5 and measurement of the p*K*_a_ values by ^1^H NMR allowed the determination of the complex stability constant of the Eu-mcp complex with log *K*_Eu-mcp_ = 12.9 (Figure 4), which is in perfect agreement with the literature data (log *K* = 13.0) obtained by from potentiometric titration.^14^

Additionally, the complex stability constant log *K* of the Eu-mcp complex was determined by ITC (Figure 4). Therefore, aliquots of a macropa solution were titrated to the Eu^3+^ solution at pH 3.5, as discussed previously and analyzed by including the p*K*_a_ values from ^1^H NMR. The complex stability was determined as log *K*_Eu-mcp_ = 13.0, which is highly consistent with both the values obtained by TRLFS and those from the literature. This technique was subsequently used for the log *K* value determination of all other selected metal ions with the macropa ligand.

**Figure 5:**
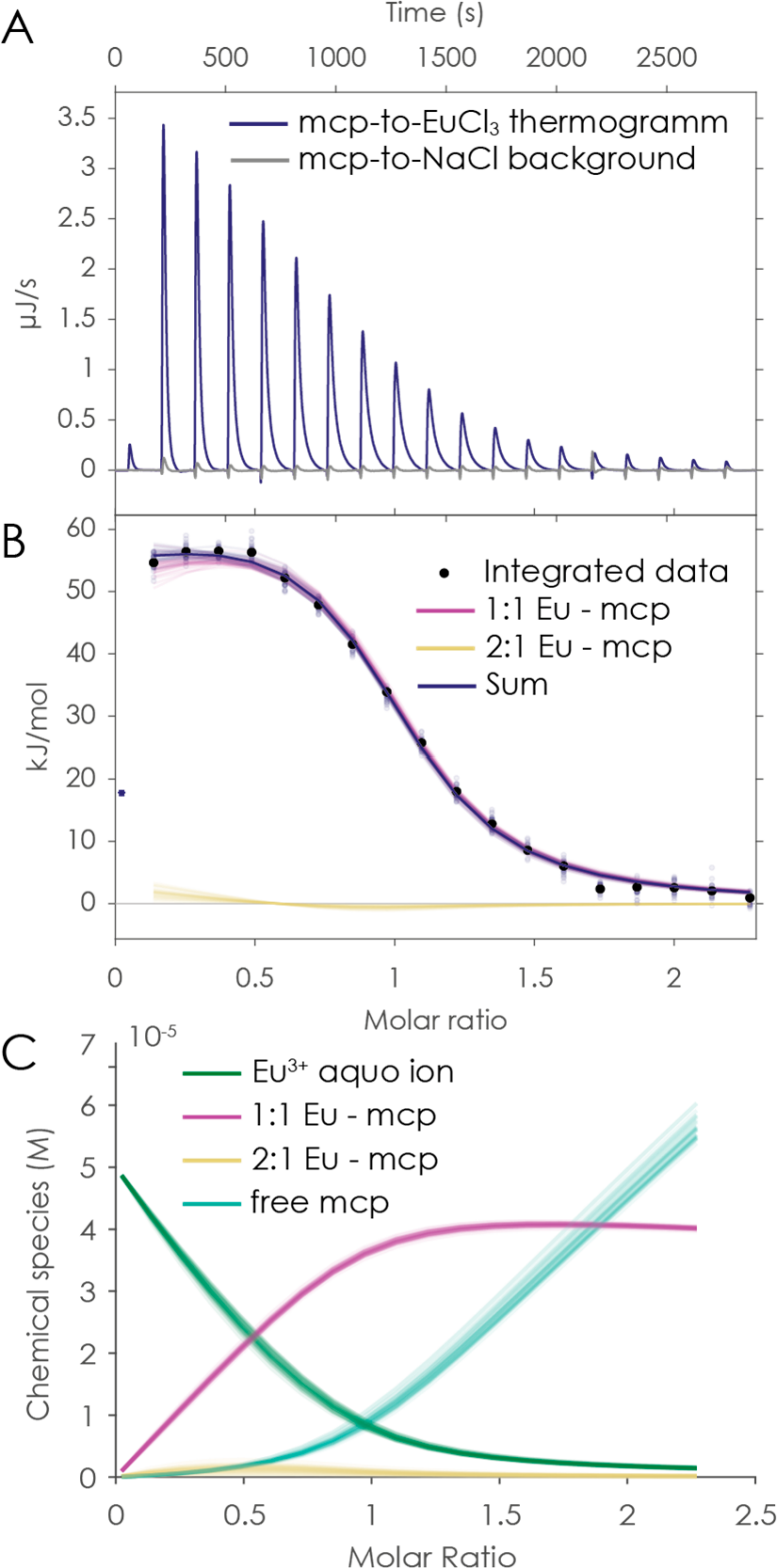
ITC results for a titration of 500 μM macropa to 50 μM EuCl_3_ in 100 mM NaCl at pH 3.6. The integrated heat (B) of the thermograms (A) was fitted with an additional 2:1 Eu^3+^-mcp complex due to comparability to lanthanum ITC (see the Supporting Information). The underlying speciation is shown in part C.

With the consistent data for the Eu^3+^ system, we generalized our approach for the selected nonluminescent metal ions Pb^2+^, La^3+^, Lu^3+^, and Ba^2+^ of radiopharmaceutical interest. Prior to the thermodynamic investigations, the identity of the formed M^n+^-mcp complexes was validated by ^1^H NMR (Figure S1) and compared with those reported.^14,25,41^ Based on the p*K*_a_ determination by NMR, the pH dependency of macropa’s affinity to different metal ions had to be fine-tuned by pH adjustment to determine the thermodynamic data. A pH value of 2 was used for Pb^2+^, whereas a pH of 3.5 was applied for La^3+^ and pH 5 was used for Ba^2+^ and Lu^3+^. By comparing the thermograms, an obvious difference between the lanthanides (Figures 5, S6, and S7) and main-group metals (Figures S8 and S9) was apparent. In the thermograms, an endothermic complexation reaction was found for the lanthanides, with heat being consumed, whereas for the other metals, an exothermic reaction was observed, with heat being released during complexation. The stripping of the hydration shell of the lanthanides compared to the complexation itself can be an explanation for this behavior.^52^ However, the expected 1:1 complex stoichiometry was verified for all of the M^*n*+^-mcp complexes. The resulting complex stability constants log *K*, considering the p*K*_a_ values of macropa, are shown in Table 2.

**Table 2:** Complex Stability Constants (log *K*) of Selected Metal Ions and the Macropa Chelator (Determined at 25 °C; Ionic Strength of *I* = 0.1 M NaCl) Compared with the Literature-Known Data Obtained from Potentiometric Titration

		log *K*	
complex	*n*	this work	literature
Pb-mcp	1.02 ± 0.01	18.5 ± 0.01	16.23^37^
La-mcp	0.86 ± 0.03	13.9 ± 0.19	14.99^27^
Eu-mcp	1.05 ± 0.02	13.0 ± 0.05	13.01^27^
Ba-mcp	1.17 ± 0.03	11.0 ± 0.10	11.11^22^
Lu-mcp	0.93 ± 0.07	7.3 ± 0.19	8.25^27^

Additionally, the respective 2:1 Ln-mcp complexes (Ln = Eu, Lu, La) needed to be considered during the initial injections of the ITC titration (high excess of metal ion) to fit the data correctly. This is especially observed in the ITC data for the La-mcp complex (Figure S7). The underlying speciation is shown in Figure 5C. However, these 2:1 Ln-mcp complexes play only a very minor role for consideration of the thermodynamic stability and no role at all for future applications in radiopharmaceuticals, where the metal is in deficit compared to the chelator.

### 3.4 Structural Investigation of the M^*n*+^-mcp Complexes

Over the past years, first DFT calculations with macropa complexes were performed on a high theoretical level.^53−55^ However, only a few metal complexes have been examined, so that general knowledge about the metal-dependent complexation of the macropa ligand is still missing. Therefore, mcp complexes with Pb^2+^, Ba^2+^, Ra^2+^, La^3+^, Eu^3+^, and Lu^3+^ were optimized to create a comparable pool of structures, with these heavy elements coordinating to the macropa ligand. In combination with information received from NMR and TRLFS, structure elucidation was possible.

Such structural information about the complex can be obtained from the Eu emission spectra. This type of information requires high-quality spectra, which are prevented by various physical effects, such as Doppler broadening. Therefore, in addition to TRLFS at 25 °C, a sample containing EuCl_3_ (10 μM) and macropa (25 μM) was measured at *T* < 40 K. Under such conditions, the splitting pattern of the ligand-field level of the ^7^F_*J*_ terms can be fully resolved. The resolution of the emission can be further increased by the choice of the used grid.

**Figure 6:**
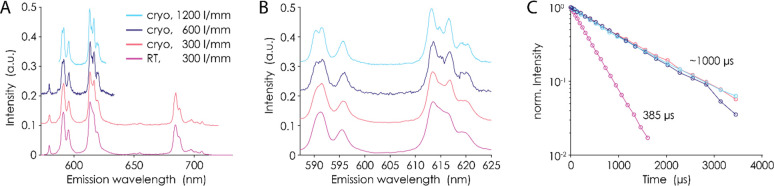
Comparison of TRLFS at 25 °C and cryo-TRLFS of the Eu-mcp complex. (A) Emission spectra collected at different temperatures and using different grids. Generally, all emission spectra provide the same features so that the complex can be assumed to be temperature-stable. (B) Zoomed representation for the comparison of the F_1_ and F_2_ bands. It can clearly be seen, by decreasing the temperature, that more details are revealed. The maximum resolution is achieved using cryogenic conditions combined with the 1200 lines/mm grid (light blue). The three- and five-times splittings are attributed to the F_1_ and F_2_ band, respectively. (C) Lifetime of the Eu emission assumed to be temperature-independent. However, in our case, the emission lifetime significantly increases to around 1 ms under cryogenic conditions.

By using the emission spectrum of the Eu-mcp complex (Figure 6), it is possible to probe the site symmetry. Especially, the *J* = 0, 1, 2 transitions of the ^5^D_0_ → ^7^F_*J*_ Eu^3+^ luminescence (Figure S3) were inspected closely and compared with the emission spectrum of the Eu^3+^ aquo ion. First of all, the ^5^D_0_ → ^7^F_0_ transition, which is symmetry-forbidden according to the Laporte selection rule,^56^ is visible in the macropa complex.

In Figure 6B, it is pointed out that the ^5^D_0_ → ^7^F_1_ transition is split 3-fold. Furthermore, a 5-fold splitting of the ^5^D_0_ → ^7^F_2_ transition is clearly visible using the best resolution grid under cryogenic conditions. The splitting patterns of these two transitions cannot be resolved under ambient conditions. The ^5^D_0_ → ^7^F_4_ transition shows the most significant change in the emission spectra. It can split up to a maximum of 9, and a low-energy *J* level of the macropa complex provides the highest probability. In summary, the splitting patterns of the ^7^F_0_, ^7^F_1_, and ^7^F_2_ transitions of the Eu-macropa complex are representative to a low-symmetry class such as C_2_ or lower.^57−59^

Additional information about the complex structure is given by the luminescence decay time of the Eu^3+^ complex. The energy transfer from the excited metal to the OH oscillators represents an efficient quenching mechanism of Eu^3+^. On this basis, an empirical equation was established [so-called Horrocks equation: *n*(H_2_O) ± 0.5 = 1.05/τ – 0.44] to estimate the number of water molecules in the first coordination sphere of Eu^3+^.^60−62^

While the determined lifetime of the Eu-mcp complex at 25 °C is quite short (∼385 μs) and suggests about two water molecules remaining in the coordination sphere, the lifetime increases significantly to ∼1 ms under cryogenic conditions. This value suggests 0 or 1 remaining water molecule and seems more realistic for the highly coordinating ligand. The standard quenching of OH oscillators should be temperature-independent. Significant changes in the luminescence decay are therefore indicative of structural changes. However, the emission spectra are fully conserved. It is likely that, because of the unique multidentate cage structure, at ambient temperatures additional quenching effects arising from, e.g., CH_2_ oscillators, add to the regular quenching mechanism from OH oscillators.

Structural analysis of the complexes using DFT calculations reveals distinct coordination characteristics for different metal ions. In the case of Ba^2+^, La^3+^, and Ra^2+^, the macropa ligand retains the coordination type observed in the crystal structures of certain M^*n*+^-mcp complexes.^14,25,41^ A total of four nitrogen atoms and six oxygen atoms are involved in the coordination between the metal center and ligand. Additionally, one water molecule is involved in the first coordination sphere. In contrast, the water molecule in the Pb-mcp complex exhibits a different behavior. Instead of coordinating to the metal center by its oxygen atom, it rather features a second-shell explicit ligand hydrogen-bonded to two oxygen atoms of the macropa ligand.

A coordination number (CN) of 10 was found for Eu^3+^ complexed by the macropa ligand, with one coordination site occupied by an explicit water molecule. Thus, unlike the complete coordination observed in the macropa complexes Ba^2+^, Ra^2+^, and La^3+^, one oxygen atom of the macropa ring is not involved. The presence of an explicit water molecule fits very well with the luminescence lifetime under cryogenic conditions. The symmetry of the DFT-optimized complex of Eu^3+^ with macropa is slightly disturbed compared to that of the La^3+^ complex and thus agrees well with the results of cryo-TRLFS. In the case of Lu^3+^, only 7 out of the possible 10 donor atoms of the macropa ligand participate in the complexation. The remaining water molecule in this complex acts as a bridge between certain heteroatoms in the macropa ring and the metal center, resulting in a total CN of 8 for the Lu-mcp complex. As a borderline cation according to the hard–soft acid–base concept, Pb^2+^ is found in complexes with CNs between 2 and 10.^39,63^ In the case of Pb-mcp, a total CN of 8 was found, which is also observed in Pb complexes of DOTA, DOTAM, or DTPA with high log *K* values of >18. One out of the four oxygen atoms and one of the nitrogen atoms of the aza-crown ether moiety found in macropa do not participate. Due to the flexibility of this ring, together with the two picolinate residues that embed the Pb^2+^, a highly stable coordination is possible.

**Figure 7:**
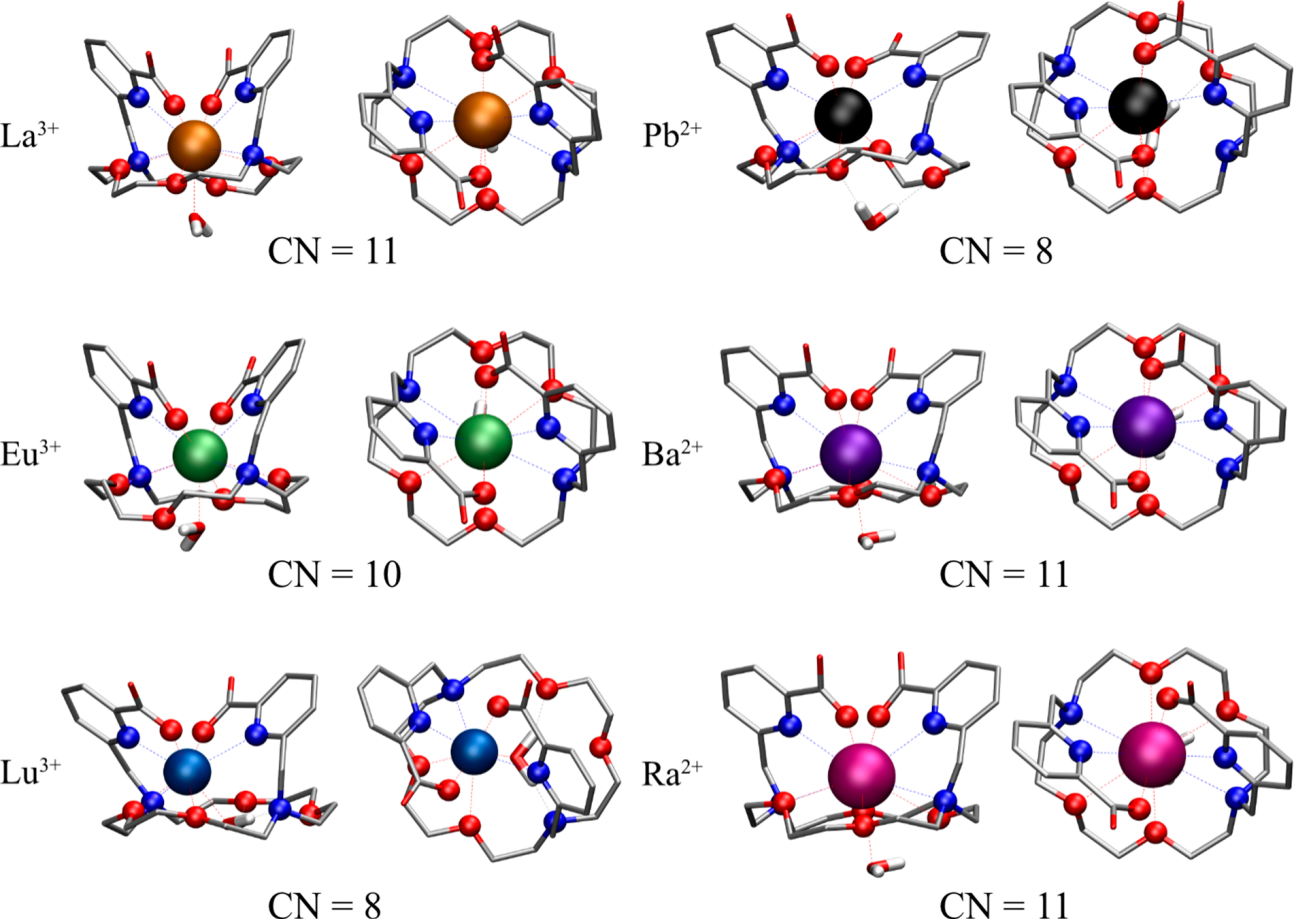
Side and top views of geometry-optimized [M(mcp)(H_2_O)]^(*m*−2)+^ complexes (Pb^2+^, Ba^2+^, Ra^2+^, La^3+^, Eu^3+^, and Lu^3+^) in aqueous solutions and the metal CNs.

**Figure 8:**
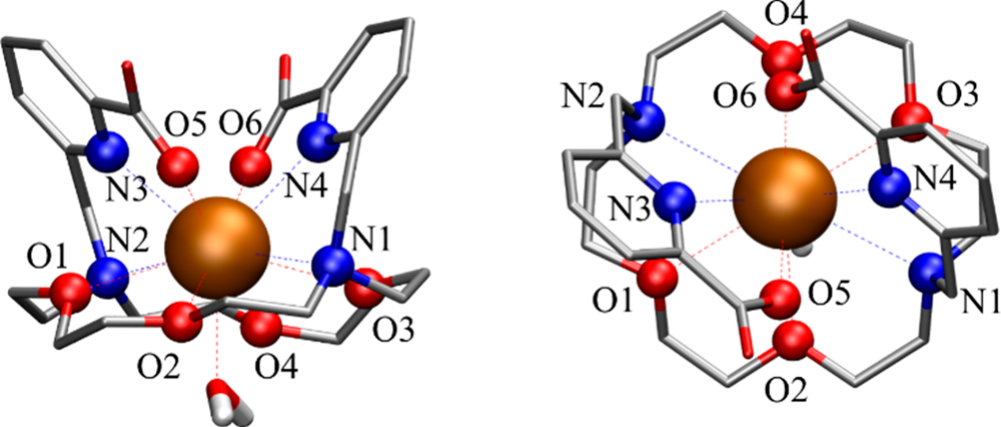
Side and top views with the atom labeling of macropa in the example of the [La(mcp)(H_2_O)]^+^ complex.

In terms of coordination with the macropa ligand, all of the complex structures appear to be similar. The metal ion is located above the plane of the macrocyclic ring system and is enclosed by both picolinic residues from above the plane. However, the CNs of the metal ions range from 8 to 11, while the hapticity of the mcp ligand ranges from η^7^ to η^10^, resulting in complex structures of different symmetry. A slightly reduced η^9^ coordination was found for the Eu-mcp complex, binding one less oxygen atom. Remarkably, the Pb-mcp complex is unique because its metal coordination is fully satisfied by the macropa ligand itself and requires no additional water molecule in the first coordination sphere. However, within the threshold of 3.4 Å defined as a coordinating bond, DFT calculations imply that one ether oxygen and one amine nitrogen of the macrocycle are not coordinating (η^8^); cf. Table 3 and Figures 7 and 8.

**Table 3:** Binding Distances M–X (Å) of the [M(mcp)(H_2_O)]^(*m*−2)+^ Complexes (M = Ba^2+^, La^3+^, Eu^3+^, Lu^3+^, Pb^2+^, and Ra^2+^) in Aqueous Solutions^a^with Atom Assignment in the Macropa Structure Shown in Figure 8

	(M−)X					
	Pb–X	Ba–X	Ra–X	La–X	Eu–X	Lu–X
O1 (mcp)	3.09	3.00	3.07	2.84	2.72	2.37
O2 (mcp)	3.29	3.01	3.12	2.92	2.75	2.89
O3 (mcp)	–	2.93	3.19	2.83	2.97	–
O4 (mcp)	3.08	3.25	2.95	2.98	–	–
N1 (mcp)	2.91	3.02	3.10	2.98	2.89	2.57
N2 (mcp)	–	3.19	3.22	2.98	2.97	–
O5 (mcp)	2.39	2.70	2.72	2.44	2.29	2.17
O6 (mcp)	2.35	2.70	2.78	2.45	2.32	2.19
N3 (mcp)	2.54	2.88	2.88	2.70	2.54	2.37
N4 (mcp)	2.71	2.85	2.94	2.70	2.57	2.66
O (H_2_O)	–	2.96	3.10	2.59	2.45	2.25
estimated total CN	8	11	11	11	10	8

a Atom pairs with distances above 3.4 Å are considered to form no coordinative bond and are marked with “–”.

Similarly, a notable decrease in coordination (η^7^) was found for the Lu-mcp complex, interacting with three less donor atoms in the macropa ligand. Additionally, the Lu-mcp complex exhibits asymmetry due to the added water molecule occupying a position between the metal center and macropa ligand, bridging the coordination. This observation arises from the small size of the Lu^3+^ ion, which prevents it from accommodating the full 10-fold coordination of the macropa ligand. Along the series of La^3+^, Eu^3+^, and Lu^3+^, i.e., for decreasing ionic radius, the complexes’ asymmetry increases with decreasing binding affinity.^14^

### 3.5 Radiolabeling

**Figure 9:**
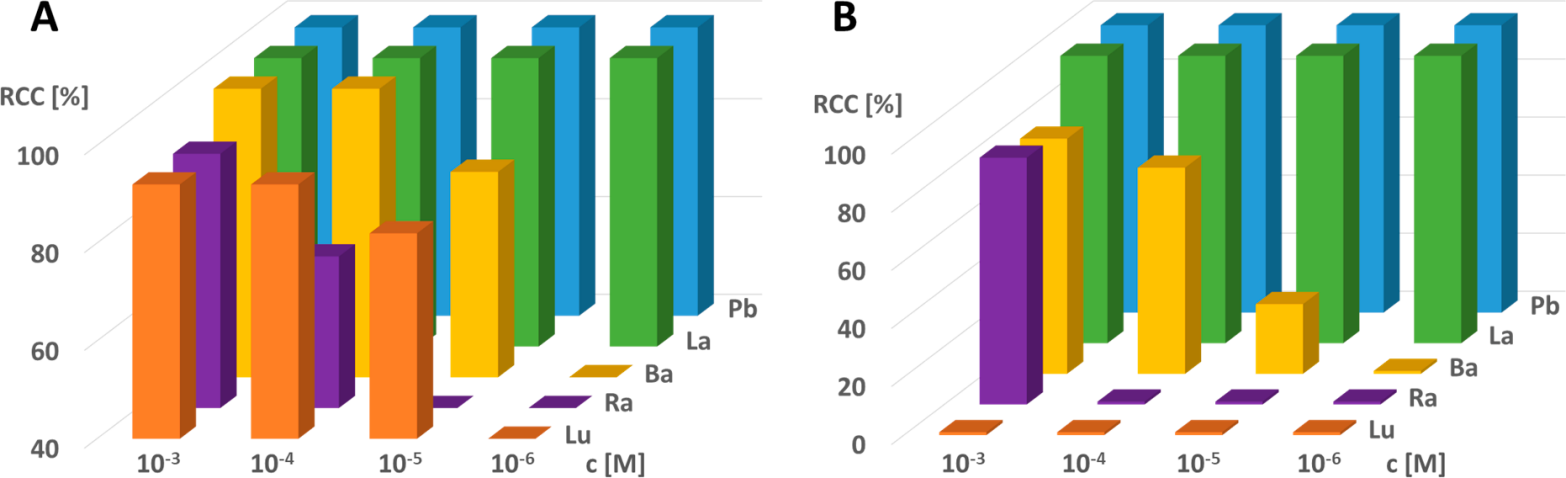
Radiolabeling of macropa with ^177^Lu (red), ^224^Ra (violet), ^131^Ba (orange), ^133^La (green), and ^212^Pb (blue) in a serial dilution of four concentrations. RCCs were determined after 1 h of reaction time at 25 °C: (A) normal phase, cyano-modified (eluent: 3:7 acetonitrile/water); (B) normal phase (eluent: 50 mM EDTA, pH 5.5).

The macropa chelator was reported as a well-working complexing agent for ^225^Ac^64^ and, to the best of our knowledge, is the best chelator for ^223/224^Ra^23,65^ so far, but it is not ideal. Moreover, there is still a lack of suitable diagnostic radionuclides for these α emitters. For this purpose and to close this gap, the production and purification of ^131^Ba^22^ and ^133^La^37^ was established. Additionally, a new theranostic radionuclide pair was recently reported with ^203^Pb and ^212^Pb^66,67^ but is mainly used in DOTA-related chelating systems.^39^ For completeness, the therapeutic β-emitter ^177^Lu^68^ was also used for radiolabeling in this study. To compare the different radionuclides, 100 kBq of ^212^Pb, ^133^La, ^131^Ba, ^224^Ra, and ^177^Lu were incubated with different concentrations of macropa (10^–3^, 10^–4^, 10^–5^, and 10^–6^ M) for 1 h at 25 °C. Two normal-phase TLC systems were used to determine the radiochemical conversion (RCC).^17^ The first TLC system was designed to monitor the radiochemical conversion of radiolabeling, whereas the second system was developed to test the stability of the complex against other complex agents by challenging the complex with a 50 mM EDTA solution. In this case, the free radionuclide moves with the solvent front (Figures S10 and S11). Concentration-dependent RCCs for the tested radiometal ions, which were calculated by using both TLC systems, are presented in Figure 9.

A quantitative conversion was found for the radiolabeling of ^212^Pb with macropa. No free ^212^Pb was observed even at a concentration of 10^–7^ M at room temperature (Figure S11). Additionally, by challenging the ^212^Pb-mcp complex with an EDTA solution, no free ^212^Pb was observed. This finding is superior over the labeling with standard DOTA derivatives,^39^ which mostly requires higher temperatures. Such mild radiolabeling conditions are highly favorable for use with temperature-sensitive biological targeting molecules such as antibodies that degrade at elevated temperatures. These results indicate that macropa provides an effective coordination environment for radioisotopes of Pb. Next, quantitative formation of the [^133^La]La-mcp complex was achieved at concentrations up to 10^–6^ M. This finding is consistent with the labeling conditions and results of a [^133^La]La-mcp-PSMA radioconjugate^37^ and of the [^225^Ac]Ac-mcp complex^17^ reported recently. [^131^Ba]Ba-mcp was previously investigated as well^22^ but showed quantitative complex formation only until 10^–4^ M. When the complex was challenged with a 50 mM EDTA solution, a major fraction of 81% intact [^131^Ba]Ba-mcp complex at a macropa concentration of 10^–3^ M combined with an increase of free ^133^Ba with 19% was observed. Furthermore, at a macropa concentration of 10^–5^ M, 24% of stable complex was still visible after the challenge. In contrast to ^131^Ba, ^224^Ra was formed with only 92% conversion at a ligand concentration of 10^–3^ M. At a concentration of 10^–4^ M, only 71% ^224^Ra was converted.^100^ These findings are comparable with the results published by Abou et al.^69^ Finally, the [^177^Lu]Lu-mcp complex was observed at ligand concentrations of 10^–3^ and 10^–4^ M with approximately 92% RCC at the chosen labeling conditions. However, the complex was challenged with a EDTA solution, only free [^177^Lu]Lu^3+^ was observed at all investigated concentrations, and no stable [^177^Lu]Lu-mcp complex remained.

## 4 Conclusion

This paper covers an analytical method workflow for the precise determination of stability constants using NMR, ITC, and Eu-TRLFS to fully characterize complexes, even at low metal-ion concentrations, which is particularly relevant for radiopharmaceutical applications. This can be used in ligand investigation to make precise predictions for work in the radiotracer range for an easy comparison of different chelating systems regarding their metal coordination behavior. To verify the whole concept, the decadentate ligand macropa was used as a chelating compound. p*K*_a_ values were determined as a prerequisite for determination of the final log *K* values. Eu-TRLFS was used to examine the Eu^3+^ speciation in the desired pH range, giving the first molecular insight by showing a 1:1 M^*n*+^/mcp ratio of the formed complexes. Subsequently, ITC measurements were accomplished to obtain the log *K* values (Pb-mcp, 18.5; La-mcp, 13.9; Eu-mcp, 13.0; Ba-mcp, 11.0; Lu-mcp, 7.3). These values were supported by theoretical calculations and are also in excellent agreement with previously published data. Although DFT calculations were not aimed at calculating the thermodynamic quantities, structural features provide clues about the relative order of experimentally determined stability constants. Finally, the radiolabeling results with five selected radionuclides perfectly underpin the obtained thermodynamic data, demonstrating the highest RCC and the highest stability with ^212^Pb even to a ligand concentration down to 10^–7^ M followed by ^133^La, which shows results comparable to those of ^225^Ac as a theranostic matched pair. Macropa is able to complex ^133^Ba, ^224^Ra, and ^177^Lu as well, however, with lower stability, and hence requires higher ligand concentrations.

The radiolabeling results nicely reflect the determined complex stabilities, proving that macropa provides an effective chelating system for ^212^Pb, ^133^La, and ^225^Ac, even under mild radiolabeling conditions, which is essential for sensitive macropa-functionalized biomacromolecules like antibodies or proteins.

## Supporting Information Available

^1^H NMR data of the macropa ligand and complexes, along with data obtained from TRLFS, ITC, and DFT calculations, as well as TLCs from radiolabeling (PDF)
