# Supplementary material for: Equilibrium Thermodynamics of Macropa Complexes with Selected Metal Isotopes of Radiopharmaceutical Interest
Source: Inorg Chem. 2023 Sep 13;62(50):20699–709. doi: 10.1021/acs.inorgchem.3c01983 (PMC10731647; doi:10.1021/acs.inorgchem.3c01983)
Supplement: Supplementary file 1 — ic3c01983_si_001.pdf [file ic3c01983_si_001.pdf]

Supporting Information

Equilibrium Thermodynamics of Macropa Complexes with Selected Metal Isotopes of Radiopharmaceutical Interest

Magdalena K. Blei[a,b,‡], Lukas Waurick[b,c, ‡] Falco Reissig[a], Klaus Kopka[a,b,d,e], Thorsten Stumpf[b,c], Björn Drobot[c], Jerome Kretzschmar[,c], and Constantin Mamat[*,a,b]*

[a] Helmholtz-Zentrum Dresden-Rossendorf, Institute of Radiopharmaceutical Cancer Research, Bautzner Landstraße 400, D-01328 Dresden, Germany.

[b] TU Dresden, Faculty of Chemistry and Food Chemistry, D-01062 Dresden, Germany.

[c] Helmholtz-Zentrum Dresden-Rossendorf, Institute of Resource Ecology, Bautzner Landstraße 400, D-01328 Dresden

[d] National Center for Tumor Diseases (NCT/UCC) Dresden, University Hospital Carl Gustav Carus, Fetscherstraße 74, D-01307 Dresden, Germany.

[e] German Cancer Consortium (DKTK), Partner Site Dresden, Fetscherstraße 74, D-01307 Dresden, Germany.

Table of Contents

^{1}H NMR Spectroscopy 2

TRLFS 3

- pH Series 4
- TRLFS Titration 5

ITC 6

Radiolabeling TLC 9

DFT Calculations 10

^{1}H NMR Spectroscopy

The macropa complexes of La^{3+}, Eu^{3+}, Lu^{3+}, Pb^{2+}, and Ba^{2+} were prepared upon mixing 1.0 equiv. (12.5 mM) macropa in D_2O with an excess of metal ion (1.2 equiv.) in D_2O at pH 6.0 (± 0.5), and warming the mixture at 40 °C for 5 min in an ultrasound bath to support complexation. Therefore, the following salts were used for complexation: $La(NO_3)_3·6H_2O$ (Sigma-Aldrich, 99.99%), $EuCl_3·6H_2O$ (Sigma-Aldrich, 99.9%), $LuCl_3·6H_2O$ (Sigma-Aldrich, 99.99%), $Pb(NO_3)_2$ (Strem Chemicals, 99.99%), $BaCl_2$ $·2H_2O$ (Sigma-Aldrich, 99.99%).

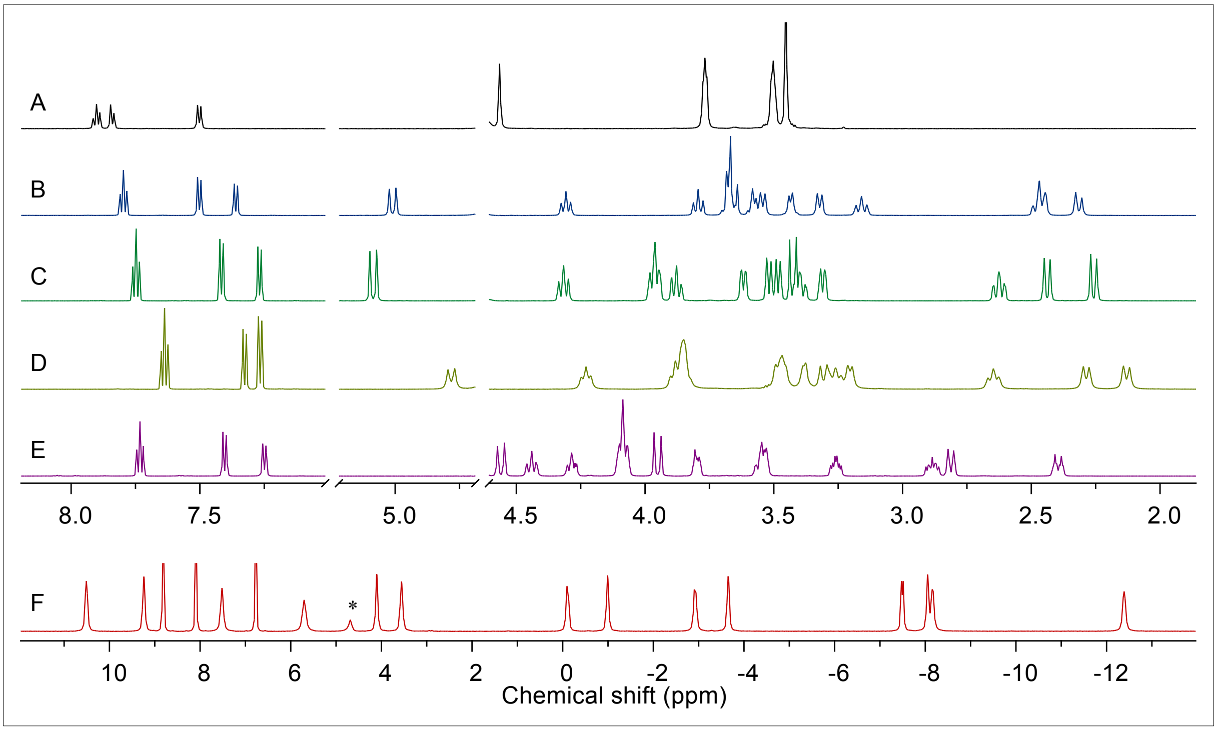

Figure S1. ^{1}H NMR spectra obtained from D_2O solutions of 1 mM mcp ligand only (**A**) as well as of 12.5 mM mcp in presence of 1.2 equivalents of Pb^{2+} (**B**), La^{3+} (**C**), Ba^{2+} (**D**), Lu^{3+} (**E**), and Eu^{3+} (**F**), at pD 6.2 ± 0.5 at (25 ± 1) °C. For clarity, only spectral regions of interest are shown. The asterisk denotes the signal from residual water (HDO). Note the different scaling of the x-axes.

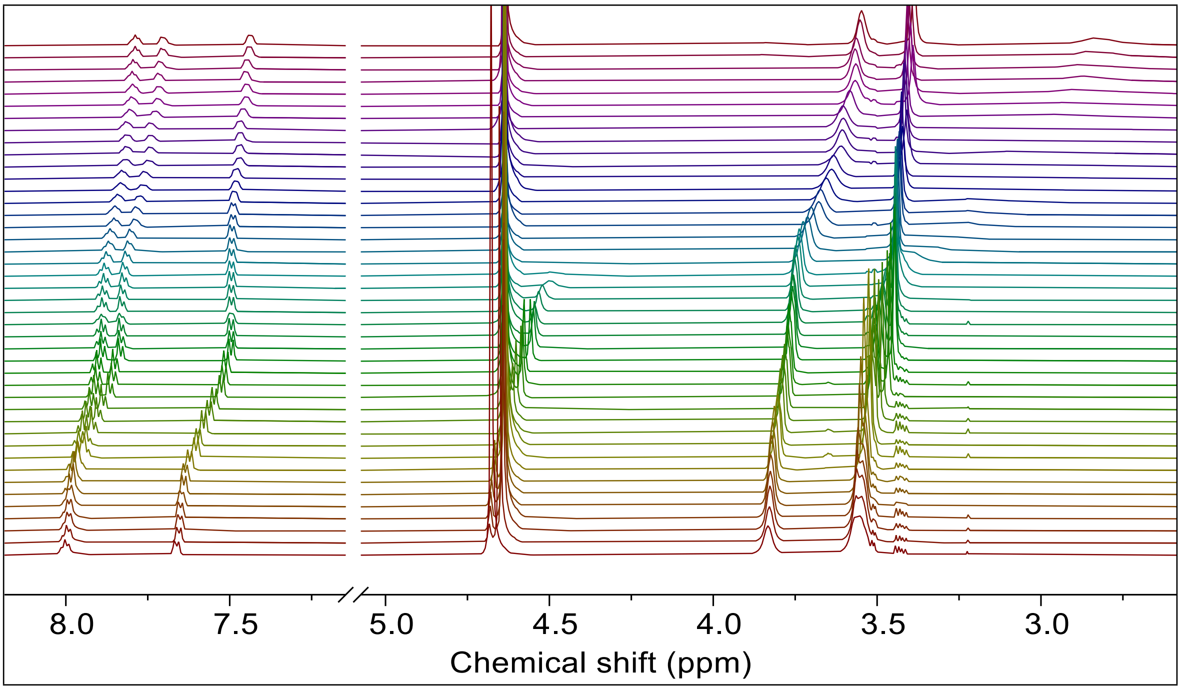

Figure S2. ^{1}H NMR pD-titration series of the ligand macropa obtained from 1 mM of ligand in 0.1 M of NaCl aqueous D_2O solutions at (25 ± 1) °C in the pD range 0.8 – 9.6 (from bottom to top). For clarity, only selected spectral regions of interest are shown. For better visualization of the broad signals, an exponential line broadening factor of 20 Hz was applied. The signal at 4.65 ppm is the residual HDO resonance, partly obscuring the benzylic methylene ^{1}H signal.

TRLFS

Table S1. TRLFS instrument setup.

linear step size	7 + 7 × *i*	µs
initial delay	12	µs
width	300	µs
slit width	200	µm
accumulations	8	
kinetic series length	21	
gain	3000	

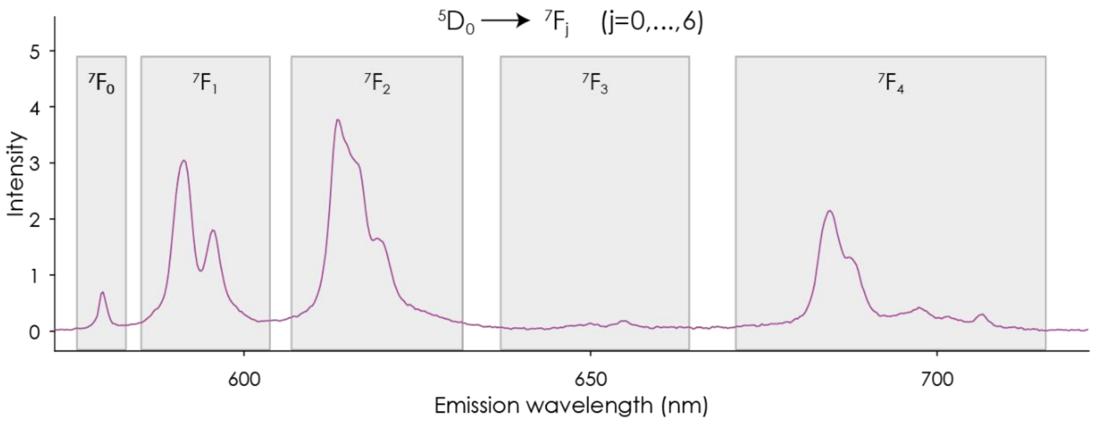

Figure S3. The transitions $^5D_0 \rightarrow {}^7F_j$ of the Eu-mcp complex.

pH Series

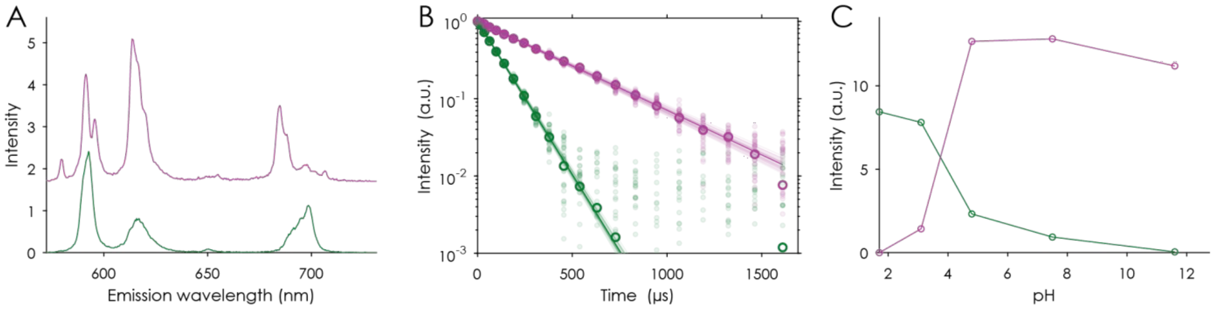

Figure S4. PARAFAC results of pH series of 10 µM $EuCl_3$ and 10 µM macropa solution in 100 mM NaCl. Emissions spectra of Eu-mcp (pink) and Eu^{3+} aquo ion (green) (**A**). Life time of Eu-mcp (pink) and Eu^{3+} aquo ion (green) (**B**) and distribution of the two species (Eu-mcp (pink) and Eu^{3+} aquo ion (green)) (**C**) at different pH values.

TRLFS Titration

Table S2. Pipetting scheme for the TRLFS titration.

V ($EuCl_3$) (µL)	V (mcp) total (µL)	V (mcp) added (µL)	c (mcp) (µM)
2000	0.0	0,0	0,0
2000	4.0	4.0	1.0
2000	5.2	1.2	1.3
2000	6.8	1.6	1.7
2000	8.8	2.0	2.2
2000	11.5	2.7	2.9
2000	15.0	3.5	3.7
2000	19.5	4.5	4.8
2000	25.4	5.9	6.3
2000	33.2	7.8	8.2
2000	43.3	10.2	10.6
2000	56.7	13.4	13.8
2000	74.4	17.6	17.9
2000	97.7	23.4	23.3
2000	129.0	31.2	30.3
2000	171.0	42.0	39.4
2000	228.1	57.1	51.2
2000	307.0	78.9	66.5
2000	418.4	111.4	86.5
2000	580.3	161.9	112.5

pH 5.5

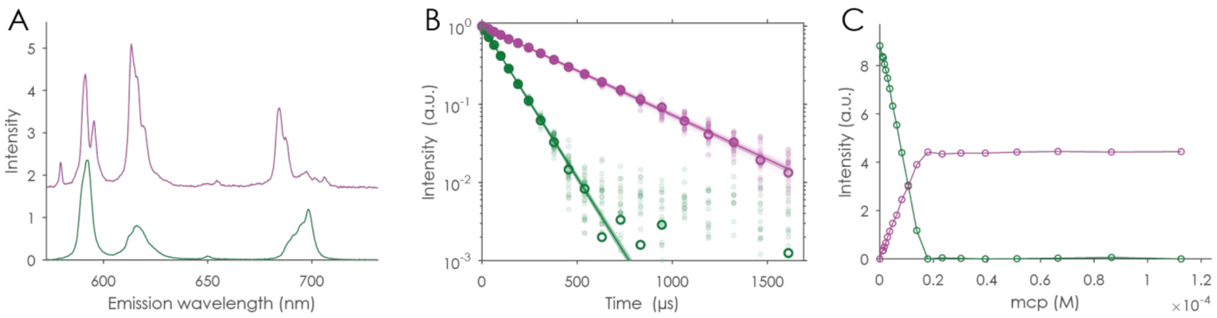

Figure S5. PARAFAC results of mcp titration (0 to 112 µM) to 10 µM $EuCl_3$ in 100 mM NaCl at pH 5.5, with a kink in the data, which do not allow thermodynamic calculations. Beside the Eu^{3+} aquo ion (green) the Eu-mcp complex (magenta) is clearly identified. Because of PARAFACs trilinearity, the emission spectra (**A**), luminescence decays (**B**), and speciation (**C**) are simultaneously determined. Transparent data points where artificially created to be used in a Monte Carlo approach for the error estimation of the underlying model.

ITC

Table S3. Instrument setup of the ITC.

Temperature (°C)	25
Reference power (µcal/s)	7.00
FeedBack	High
Stirring speed (rpm)	750
Initial delay (s)	60
Number of injections	19
Concentration syringe (µM)	600
concentration cell (µM)	50

Table S4. Injection setup for the ITC measurement.

Injection	Volume (µL)	Duration (s)	Spacing (s)
1	0.4	0.8	150
2 – 19	2.0	4.0	150

Table S5. Summary of the ITC data.

		n	log *K*		ΔH	
			1:1	2:1	1:1	2:1
**La**	global fit of a set of 3	0.861	13.87	24.88	2.553	-2.537
**Eu**	global fit of a set of 3	1.046	13.037	24.29	61.334	32.907
**Lu**	global fit of a set of 3	0.932	7.275	10.91	27.49	355.2
**Pb**	global fit of a set of 3	1.02	18.52		-46.21	
**Ba**	global fit of a set of 3	1.17	9.624		-40.72	

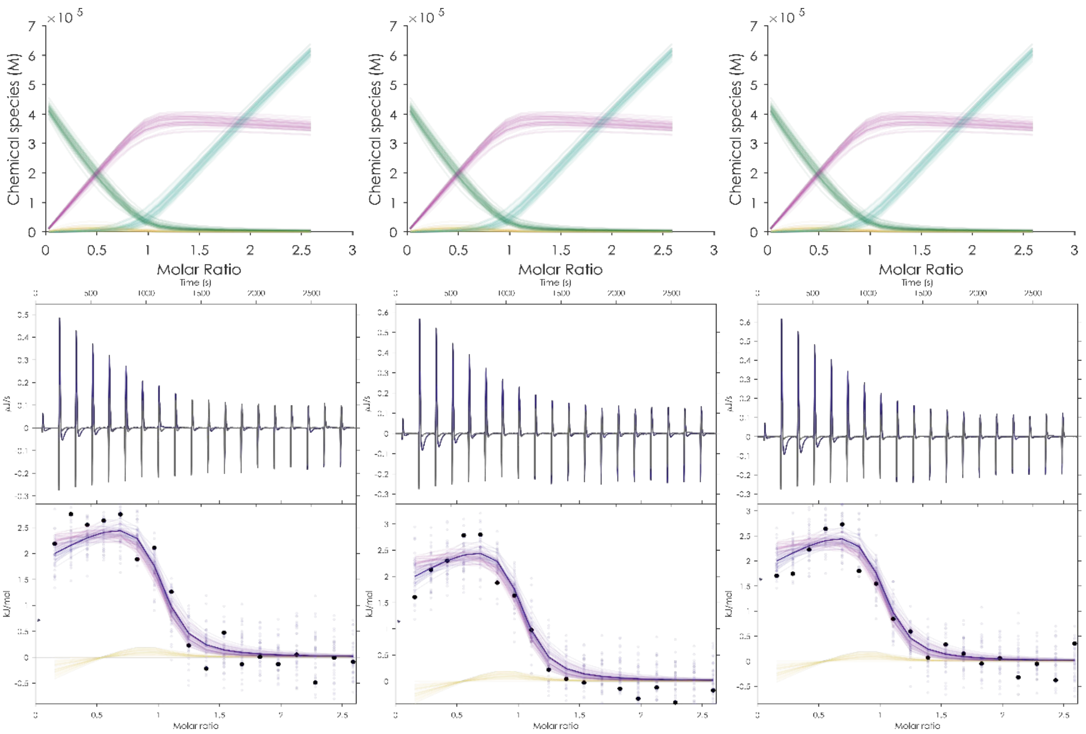

Figure S6. ITC data of the three measurements of 600 μM mcp to 50 μM $La(NO_3)_3$ solution in 100 mM NaCl. On top the speciation of the four species: La^{3+} species (green), 1:1 La-mcp species (magenta), 2:1 La-mcp species (yellow) and free mcp (turquoise), and below the fitted data in combination with the speciation of the La-mcp complexes.

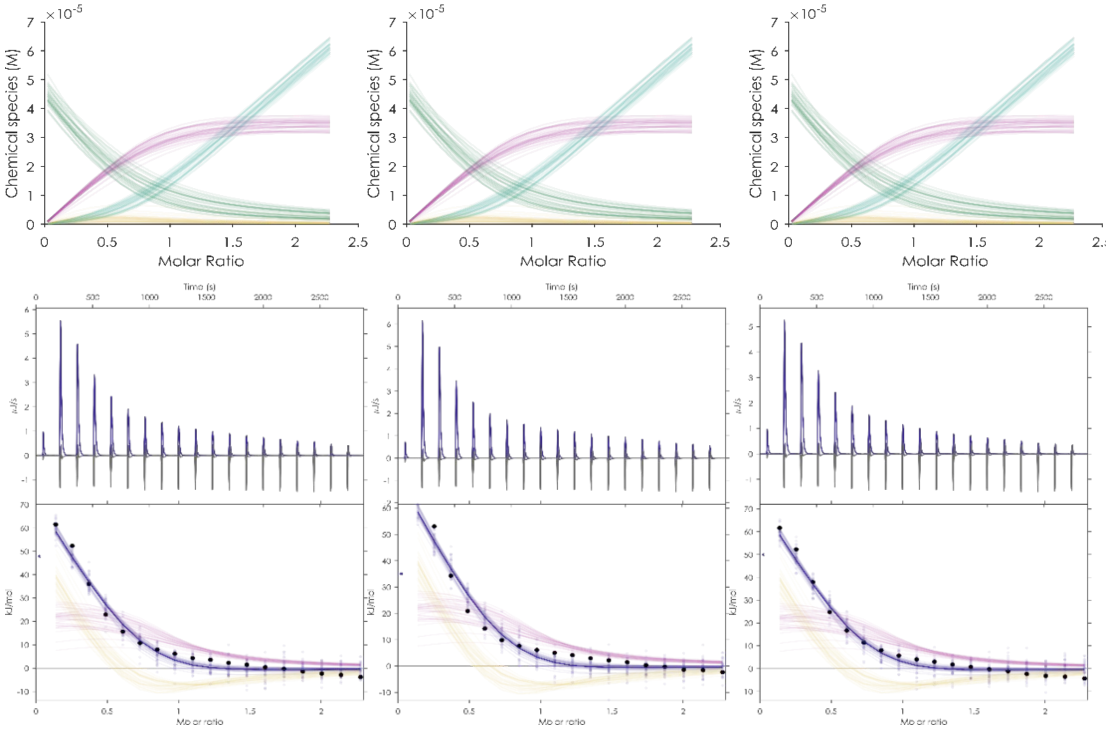

Figure S7. ITC data of the three measurements of 600 μM mcp to 50 μM $LuCl_3$ solution in 100 mM NaCl. On top the speciation of the four species: Lu^{3+} species (green), 1:1 Lu-mcp species (magenta), 2:1 Lu-mcp species (yellow) and free mcp (turquoise), and below the fitted data in combination with the speciation of the Lu-mcp complexes.

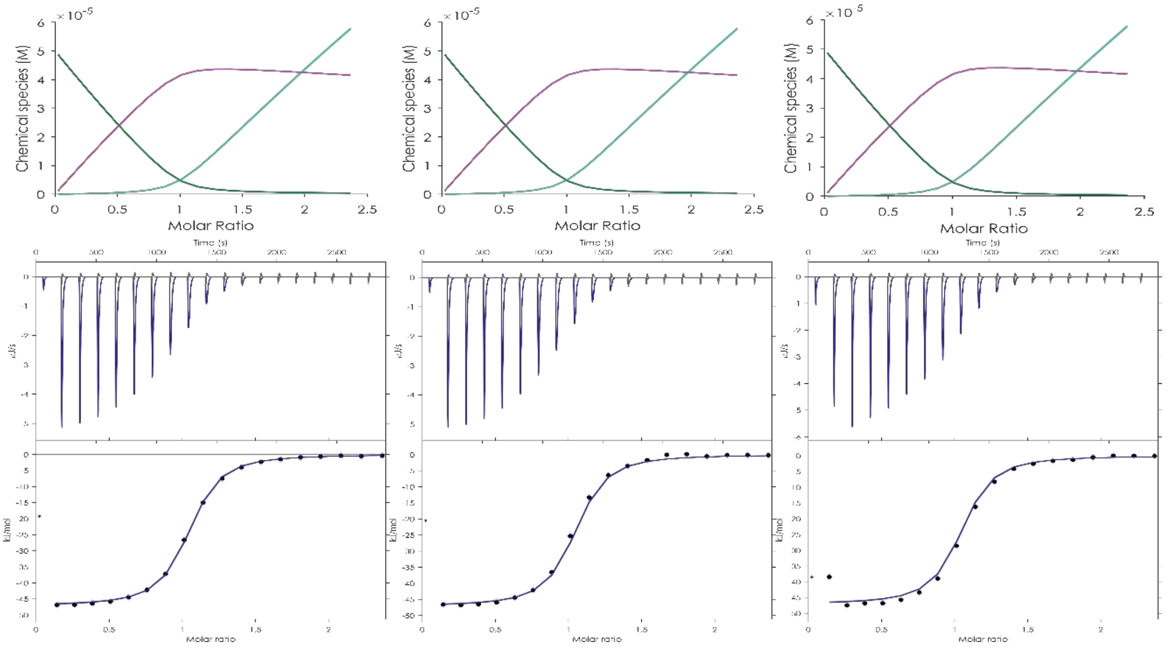

Figure S8. ITC data of the three measurements of 600 µM mcp to 50 µM $Pb(NO_3)_2$ solution in 100 mM NaCl. On top the speciation of the four species: Pb^{2+} species (green), 1:1 Pb-mcp species (magenta), and free mcp (turquoise), and below the fitted data in combination with the speciation of the Pb-mcp complex.

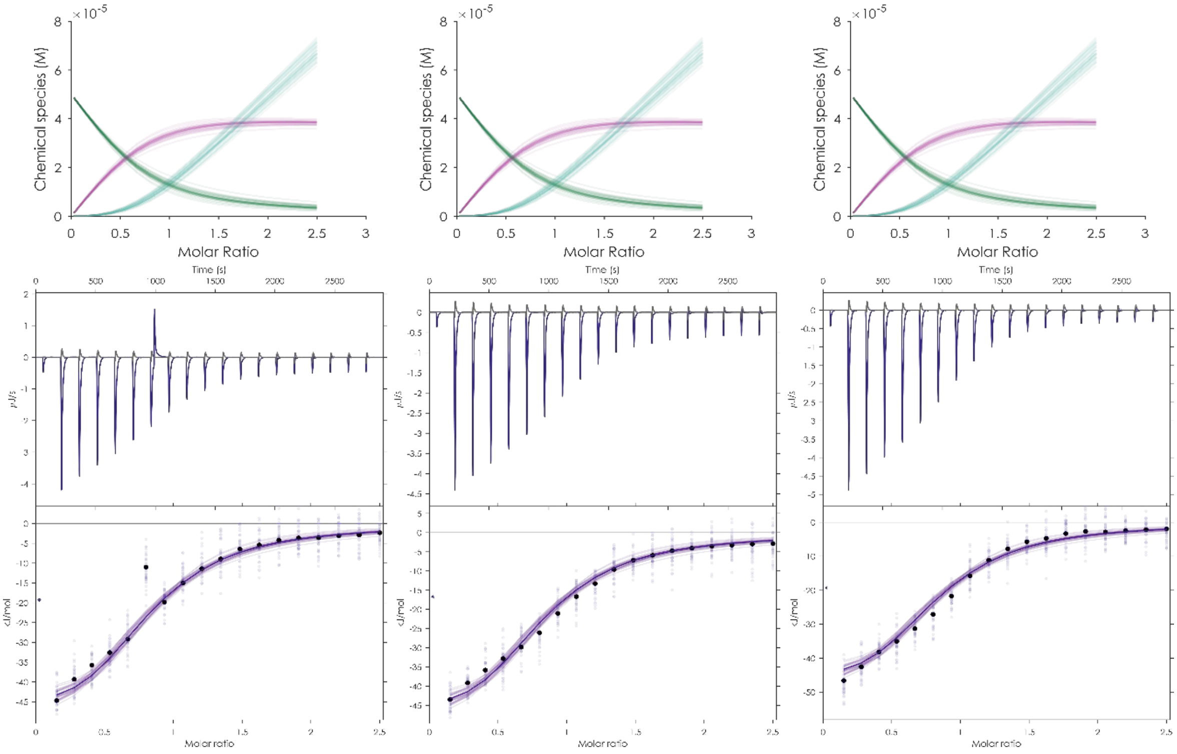

Figure S9. ITC data of the three measurements of 600 µM mcp to 50 µM $BaCl_2$ solution in 100 mM NaCl. On top the speciation of the four species: Ba^{2+} species (green), 1:1 Ba-mcp species (magenta), and free mcp (turquoise), and below the fitted data in combination with the speciation of the Ba-mcp complex.

Radiolabeling TLC

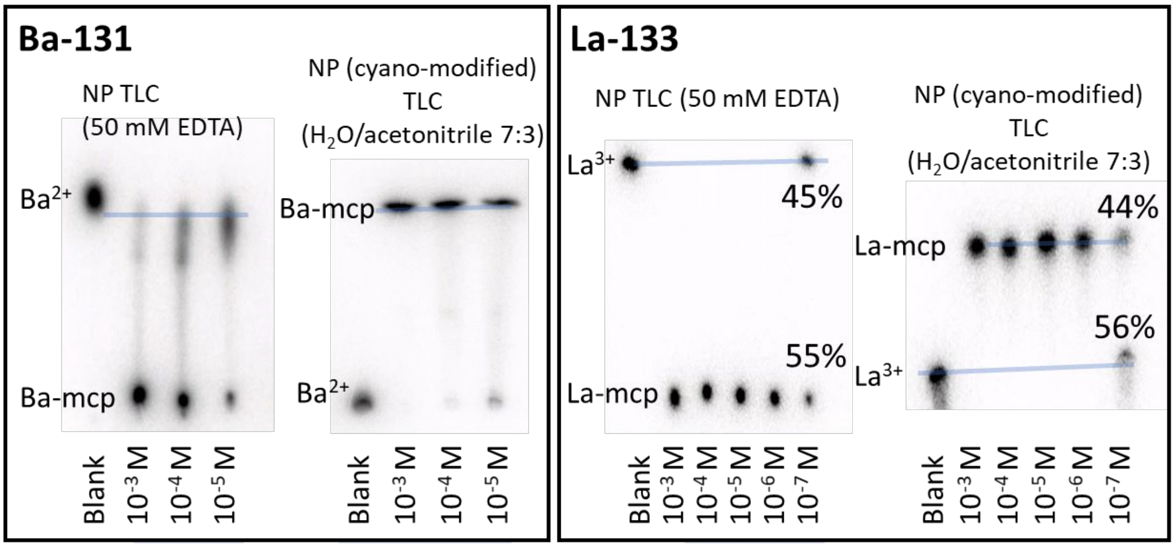

Figure S10. Radio-TLCs of macropa complex with ^{131}Ba and ^{133}La.

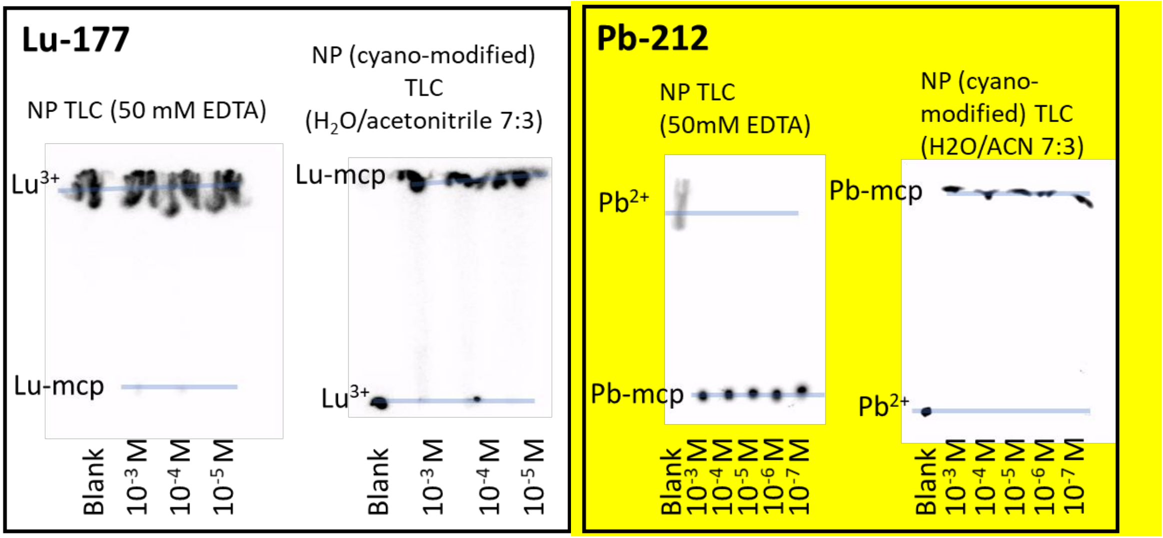

Figure S11. Radio-TLCs of macropa with ^{177}Lu and ^{212}Pb.

DFT Calculations

Coordinates (xyz) of the optimized [M(mcp)(H_2O)]$^{(m-2)+}$ structures:

M = Ba^{2+}

O	-2.68656513730567	-0.03773649179865	-1.17262997445453
C	-1.92983679700762	0.57609428828812	-0.15006877835995
C	-0.67944638690726	-0.22134787710071	0.07034179066906
H	-2.50766339382567	0.64678663079468	0.78001225264713
H	-1.64229675481113	1.59030758019042	-0.45045171540190
O	-0.99754301063004	-1.48188268150808	0.62746389137227
H	-0.01818683842976	0.32831932607882	0.75116138140455
H	-0.15521049349212	-0.35564381871967	-0.88353916352634
C	0.15024319329181	-2.29610581596786	0.78292149809891
C	-0.16639558628688	-3.42963699493979	1.71975163962343
H	0.96046443771849	-1.70837314199778	1.23037536593038
H	0.49580019262048	-2.63736924490681	-0.19960876436544
N	-1.15752331263711	-4.39005740787138	1.22846014041990
H	-0.55079353669295	-2.98726639539700	2.63986662417725
H	0.77682768470357	-3.93585348086315	1.97900271746644
C	-1.70516910337380	-5.12048624298645	2.37310170277348
C	-3.03529163867101	-5.79141081379591	2.15723144043741
H	-1.83211618929771	-4.40700627273355	3.18953534175648
H	-0.99581040700332	-5.88516697344457	2.72706297559151
O	-4.02612081576391	-4.82028100914315	1.87197655795797
H	-3.30043631415417	-6.32510247770754	3.07855257945352
H	-3.00591945577960	-6.52903335844905	1.35125358868046
C	-7.02689946891172	-2.71774902018862	-0.14426883840536
C	-6.66112467919839	-1.27304618805729	-0.36385031700045
N	-5.52645872498415	-1.02720396602493	-1.25881768108919
H	-6.40720423049015	-0.85231303236287	0.60999353940408
H	-7.55982758243166	-0.74385425934058	-0.71968349299120
C	-5.09176446968305	0.35590440341743	-1.04729466241494
C	-3.76519272527362	0.74000731072188	-1.66032511537303
H	-5.01932936101417	0.50809758750039	0.03170458354418
H	-5.85277843029679	1.06121981366889	-1.42073119061144
H	-3.59159945335944	1.79903779581080	-1.44419552801916
H	-3.77526822450176	0.63123029730121	-2.74439603991974
C	-5.93298901325343	-1.28480802250765	-2.63486673719140
O	-5.94347191568873	-3.41265034620033	0.44274387458869
H	-7.32361389252536	-3.22588480570588	-1.06861097256711
H	-7.89149706765353	-2.73052860005858	0.52692117311253
C	-6.33127061974001	-4.39608272926373	1.37812380069726
C	-5.25301320264688	-5.42920101827358	1.51783697409443
H	-7.23983183792477	-4.91080443682135	1.04713463995380
H	-6.54267896049800	-3.92202789133749	2.34483766159981
H	-5.12446503874770	-5.97583564986684	0.57620725336972
H	-5.55772764036128	-6.14616673313339	2.29006850622615
C	-4.83695688866925	-1.38627425709430	-3.65611665502997
H	-6.65969067846638	-0.52983916377142	-2.97233014417165
H	-6.45470618052651	-2.24542436681717	-2.64907742124757
N	-3.68069291495615	-1.92650244511546	-3.29625704429828
C	-5.08005955033430	-0.98293958615823	-4.96030716130663
C	-4.09518994311177	-1.15609625792060	-5.90950063132132

C	-2.71555896747273	-2.06925383220643	-4.20022662381512
C	-2.88667016951776	-1.70020927391015	-5.52217295620987
C	-1.38582813936557	-2.64807874988845	-3.72758809817355
O	-0.49474368285780	-2.77480251063618	-4.58596578816867
O	-1.29789813701178	-2.94099546670171	-2.51027410096686
H	-6.03380641547602	-0.54240201755215	-5.22212124333432
H	-4.26306972884721	-0.85820332379680	-6.93743984225561
H	-2.07445407640700	-1.84563201144239	-6.21949595711290
C	-0.50518034038731	-5.28072996012565	0.27321783880472
H	0.01529266942691	-4.65596943206428	-0.45784625455456
C	-1.39765369650382	-6.20133651693242	-0.50461362350652
H	0.26267817477773	-5.88476836914614	0.77982359178997
C	-1.03537850951152	-7.52686659173818	-0.67580162724533
N	-2.48323011333219	-5.69128787724116	-1.07695286210934
C	-3.25098583835664	-6.47541757429575	-1.82896887786493
C	-1.82097311054966	-8.34225076356919	-1.46439756192982
C	-2.95077733327802	-7.80861519090142	-2.04793247206310
H	-0.14368003080290	-7.90830743795739	-0.19530656658692
C	-4.49380829331827	-5.85427142626141	-2.45845985304939
H	-1.55724679977117	-9.38193742329079	-1.61601822649612
H	-3.60567669931562	-8.39825374310981	-2.67285332391986
O	-4.71640262805487	-4.65053010895092	-2.19181779427717
O	-5.18371632317336	-6.59582406468769	-3.18175166608320
Ba	-3.27102017260077	-2.94703118081605	-0.67184148786170
O	-3.66562486094391	-1.91230275463611	2.07489400441656
H	-3.80649726168222	-2.82056933057529	2.37353246423971
H	-2.75968695668120	-1.70171612598647	2.31885364234995

M = La^{3+}

O	-2.81095501811208	-0.20510437763637	-1.01292470131851
C	-1.90793329312332	0.53539823779003	-0.20815051660844
C	-0.67288226755953	-0.28418107644763	-0.00936331146203
H	-2.37236932847200	0.78618763615230	0.75223367509136
H	-1.62854637030708	1.46473638490079	-0.71297302710030
O	-1.01106414776178	-1.48892192202356	0.65357837298249
H	0.04544485656940	0.28283330451195	0.59400057859226
H	-0.21363815974277	-0.51595129219005	-0.97682858699535
C	0.14355694303799	-2.28305782625922	0.86933954879003
C	-0.23120360615853	-3.44998373968515	1.73337683214264
H	0.90014236727256	-1.69016739009232	1.39599516676340
H	0.56198446177264	-2.58404690673823	-0.09626826844663
N	-1.19987820853217	-4.37120652879409	1.11784331052013
H	-0.67958577837903	-3.05519745798204	2.64628364049740
H	0.68211171930259	-3.98699940772739	2.02554627464697
C	-1.77357617077332	-5.18415994983894	2.20137770274340
C	-3.06115782315783	-5.88574389513901	1.86958815891267
H	-1.98111472063705	-4.51095056406996	3.03420132324250
H	-1.04471462913184	-5.92757912884127	2.55401732985394
O	-4.00217114798267	-4.90681328689226	1.46753537185852
H	-3.41626567206151	-6.37942344416734	2.78051190774763
H	-2.95106381024838	-6.65400283092411	1.09945780833698
C	-7.01765027541148	-2.74319109088890	0.06932482009050
C	-6.77241659847859	-1.33478756971124	-0.38801493034969
N	-5.56398294685186	-1.16637804361779	-1.21606216862166
H	-6.65425919966110	-0.72088155621477	0.50539322346479
H	-7.65796413362069	-0.96429503448645	-0.92212769058826

C	-5.17491827526919	0.24885342772514	-1.11710319710318
C	-3.80830710618404	0.57938402872480	-1.65528075300819
H	-5.18446347428783	0.51033630579700	-0.05772618739852
H	-5.91646341089846	0.88739953779932	-1.62014932826204
H	-3.62308155725116	1.64068457529531	-1.47390364364245
H	-3.72995964656691	0.41608402299059	-2.73080389776773
C	-5.90072631729072	-1.54647619379084	-2.59113720102846
O	-5.94519958100114	-3.10717367632209	0.92351501184912
H	-7.09215479760565	-3.45138410465685	-0.76100578704216
H	-7.95933189611008	-2.76192316753483	0.62674997914892
C	-6.21954317511378	-4.19960299239468	1.78938413908836
C	-5.34399748344968	-5.37151218738780	1.45335234651426
H	-7.27115793718106	-4.48956094547326	1.72828794640501
H	-6.01130621194954	-3.87065842987062	2.81167706005569
H	-5.58519187159197	-5.78752697036714	0.47170602443337
H	-5.48014486255539	-6.14934524278591	2.21090224162368
C	-4.73507582480526	-1.72933248254938	-3.51910148051965
H	-6.59744001610882	-0.81826123062368	-3.02821196261241
H	-6.42316703061032	-2.50539793408507	-2.54889283165301
N	-3.59053677186004	-2.18070443979504	-3.01407227236316
C	-4.87535778293277	-1.46618670744889	-4.87846139185479
C	-3.79912227749247	-1.68266685752676	-5.71966526906561
C	-2.54024982221631	-2.36022460845335	-3.81761006245684
C	-2.60293229331259	-2.13149101474056	-5.18093242951926
C	-1.25647346357433	-2.80606750255423	-3.14625480009601
O	-0.26015441225111	-3.00617384763625	-3.84213804283573
O	-1.32139641961391	-2.92618910369297	-1.88335362975676
H	-5.81621530678044	-1.08834744965610	-5.25880903227091
H	-3.88667830520949	-1.48787979770261	-6.78187524063796
H	-1.72437429662653	-2.29613882151270	-5.78911302195820
C	-0.49682301875527	-5.20656230038504	0.14009805946211
H	0.10602827547312	-4.55096393655869	-0.49227623587926
C	-1.38771166240545	-5.99155333061266	-0.77643457143117
H	0.19327713354587	-5.89215334492393	0.64979868972363
C	-0.98567963509377	-7.23337828294738	-1.25768639929574
N	-2.54619511857386	-5.44727377156857	-1.13763211754573
C	-3.35574074934329	-6.11199688185893	-1.96355364503120
C	-1.80893482957177	-7.91156179465622	-2.13866829733683
C	-3.02244390476759	-7.34482783889801	-2.49735491806908
H	-0.04285195889056	-7.65592181209706	-0.93326579678024
C	-4.68248039113341	-5.44475813413599	-2.26327939159107
H	-1.51491617009742	-8.87835744712840	-2.52954424257314
H	-3.71118560558610	-7.83923133900654	-3.16816267219613
O	-4.89916699211696	-4.37127907559103	-1.61754055585965
O	-5.44681691062983	-5.97105033297386	-3.07210452073402
La	-3.29790021432540	-2.94310005916258	-0.44420409160948
O	-3.63303504390250	-1.63112750684079	1.76875707489554
H	-4.37295580666128	-1.83162851135914	2.34861711752070
H	-2.88408261125381	-1.39747303205243	2.32476158327869

M = Eu^{3+}

O	-2.69757429745030	-0.23099445678789	-1.12283327615993
C	-1.89636115227830	0.50696058683063	-0.21146281700143
C	-0.62732167591911	-0.25126660309307	-0.00362829330323
H	-2.42851703056453	0.64442750888233	0.73577865327168
H	-1.66210066046818	1.48909809775296	-0.63145861098367

O	-0.95448796851383	-1.51813394458551	0.53382955492345
H	0.01657826083185	0.29478280410077	0.69434978286046
H	-0.09297363268087	-0.37470313674219	-0.95253066425201
C	0.20414690337141	-2.27875306237589	0.82904760643453
C	-0.21944777078737	-3.40480823054570	1.72145020258425
H	0.92680349457675	-1.65488881168069	1.36569435382873
H	0.67089933919746	-2.61516356316233	-0.10237562441040
N	-1.20492678136528	-4.29864935389394	1.09101745389337
H	-0.67923545220526	-2.96759747420802	2.60864773335193
H	0.66172211515610	-3.96980551080918	2.05353317202801
C	-1.87883072449298	-5.02663846684149	2.18102505359140
C	-3.14355663908410	-5.75875079936257	1.82335586315822
H	-2.14393974501101	-4.28691556381190	2.93778260689563
H	-1.18234459941435	-5.73930949505004	2.64538692434554
O	-4.08276738320491	-4.82555665000630	1.32767574195722
H	-3.52245515884916	-6.21180101346125	2.74636764735537
H	-2.98415140569253	-6.56881925400945	1.10487207534887
C	-6.95698869813225	-2.80143038828550	0.21549312331145
C	-6.67714766579090	-1.41850093455340	-0.31200361854489
N	-5.45581922921029	-1.25933973265523	-1.13640122889053
H	-6.59153577390267	-0.75847710359098	0.55244144250431
H	-7.54676270010576	-1.07076391445001	-0.88416042000932
C	-5.07980819468537	0.15916094639306	-1.02228101510213
C	-3.77310540379402	0.52660004480630	-1.66897425535648
H	-5.00507106631626	0.38777881111824	0.04187302051512
H	-5.86948553570417	0.79951328219683	-1.44350623991212
H	-3.59492475016278	1.59107042296246	-1.50026157693359
H	-3.77739647795105	0.36998072878014	-2.74672638719149
C	-5.81545885947092	-1.60591536667762	-2.51611437448104
O	-6.18197178494634	-2.98938857002385	1.39467016621384
H	-6.72444442549417	-3.57104673784450	-0.52368144397487
H	-8.01739473232247	-2.87474552821590	0.48092652464794
C	-6.30471322906496	-4.27146019533996	1.98474572862902
C	-5.42285218338380	-5.29686593836370	1.31689843032577
H	-7.34934436651648	-4.60125194037957	1.96005972437878
H	-6.00411596855558	-4.15288689889094	3.02755869093743
H	-5.73341854691596	-5.48199759752120	0.28671067357539
H	-5.50235716682837	-6.23722593685185	1.86951018328649
C	-4.68152110096111	-1.76284494761151	-3.48986639624714
H	-6.52165500854024	-0.86619395801026	-2.91823142374500
H	-6.33671230550121	-2.56491667891853	-2.48801895447862
N	-3.49165337645157	-2.15978326652363	-3.04612800561964
C	-4.90044858720491	-1.52826165843258	-4.84464488431358
C	-3.86262765846942	-1.71142480983236	-5.73967878977693
C	-2.47839476157098	-2.30975224342970	-3.90303025714118
C	-2.62039700077239	-2.10155687918431	-5.26207153052919
C	-1.15425525436210	-2.71658455602398	-3.29265497252990
O	-0.16788557670691	-2.82815382683645	-4.01439047678976
O	-1.18156353615442	-2.90935132112152	-2.03076892444485
H	-5.87541065500020	-1.19770591321605	-5.18055249689843
H	-4.01619334997243	-1.53821327813434	-6.79798193050418
H	-1.76986844293736	-2.24047038407486	-5.91481744006979
C	-0.48284971515610	-5.21050588351810	0.19834865925906
H	0.21192996242232	-4.61972747898592	-0.40405658776763
C	-1.37020340089089	-5.95997039433210	-0.74422964429913
H	0.12063892220645	-5.92133924050438	0.77817581733173

C	-1.03683173761014	-7.23441215248798	-1.19064199420443
N	-2.48428359772122	-5.35358608700422	-1.13993178460129
C	-3.33379420012108	-6.00042509602595	-1.93935236387117
C	-1.88994267286481	-7.88485705232310	-2.06364449314601
C	-3.07346897474443	-7.26370101327998	-2.43602639900765
H	-0.12611212739181	-7.70402502068554	-0.84036800524926
C	-4.62624600645456	-5.26989886673280	-2.20621434893650
H	-1.64851966789517	-8.87586819101089	-2.42874542247438
H	-3.79428708875244	-7.74151388699275	-3.08502652233525
O	-4.72866369544286	-4.15818034385407	-1.59184026716790
O	-5.47533948327006	-5.77378756599346	-2.93689421485512
Eu	-3.02303881776763	-2.88896148454643	-0.66134606236730
O	-3.76480713127817	-1.81167768804560	1.41325851659196
H	-4.64136503521828	-2.20698676451379	1.61189800641574
H	-3.22515999334030	-1.89643442756445	2.20436150612577

M = Lu^{3+}

O	-2.62177104440589	-0.27199437589078	-1.18805785887060
C	-1.39152960873870	0.35118636879788	-0.81832279812846
C	-0.34646164070330	-0.70365869859533	-0.60355406770977
H	-1.55955174264443	0.92073983816619	0.10010172221679
H	-1.07127475538405	1.03245517435078	-1.61161219359323
O	-0.87997346781869	-1.68943055227614	0.26963334642455
H	0.52946553109728	-0.22506266976042	-0.15694200941556
H	-0.04582755077462	-1.17145470968620	-1.54434885799443
C	0.10191097457907	-2.22635714885589	1.14337824467892
C	-0.51964270930417	-3.12500615275743	2.18610257568003
H	0.59657638813532	-1.40713423318451	1.67591168504907
H	0.87473058915106	-2.74135281577855	0.56250719465862
N	-1.39832993938070	-4.18012341205536	1.66201325984752
H	-1.11682580275995	-2.52170734111453	2.87303912072743
H	0.30969900250082	-3.55715725466036	2.76528459761523
C	-1.86012873752613	-5.00499682445643	2.79053695771116
C	-3.04417159408184	-5.90585104090727	2.51361424125866
H	-2.13629196778062	-4.32072664894843	3.59621796769954
H	-1.03488718569760	-5.62865326758808	3.16219593521968
O	-4.19779032412992	-5.13341967936674	2.25999539302756
H	-3.19767661767594	-6.53847574370251	3.39756840733459
H	-2.85002778690309	-6.57683126772400	1.67156413463139
C	-6.81955314377576	-3.04772224325472	-0.14509484214670
C	-6.39846298270074	-1.59889077663132	-0.24936555435699
N	-5.33324984413743	-1.21917567597725	-1.22034185706763
H	-6.06063878024901	-1.28984453209166	0.74016044095351
H	-7.29372286714646	-1.00764303846084	-0.47865793424844
C	-4.93753769448691	0.15932663128037	-0.84727390536293
C	-3.66827779316667	0.65385264568961	-1.48617922954207
H	-4.77977783257273	0.16588634875848	0.23182295215131
H	-5.75081720762820	0.86034185924136	-1.07145990870939
H	-3.42764256909212	1.62385601970471	-1.04671439966036
H	-3.73599761016773	0.78186085089216	-2.56774651144239
C	-5.91622469222205	-1.24386606672737	-2.57639914228452
O	-5.84713946012744	-3.80444533555481	0.55388697642982
H	-7.01201255671518	-3.51012964133549	-1.11801311981841
H	-7.75794698288976	-3.04999362398486	0.41971326070765
C	-6.35312087115181	-4.91652813762233	1.26926435688663
C	-5.21615915224887	-5.84861499587864	1.59612102265219

H	-7.08416416169995	-5.46454674007194	0.66512805087446
H	-6.85136574131719	-4.56723439339691	2.18142289698089
H	-4.81697840355780	-6.28911658055977	0.67406021030622
H	-5.59930092589009	-6.66571206913926	2.22137010441209
C	-4.91094487805697	-1.24144201389010	-3.69110548443434
H	-6.62224231773570	-0.41481025520144	-2.70116234432010
H	-6.48430799451591	-2.17124575474463	-2.67637756283753
N	-3.70716683163579	-1.71939684367243	-3.40961719022678
C	-5.23214379334125	-0.83932664129669	-4.98320732898871
C	-4.27665272557025	-0.96510437597051	-5.97732231120463
C	-2.77998507054513	-1.84771489763728	-4.35938011280025
C	-3.02433750071236	-1.48149076968336	-5.66801878022698
C	-1.47758276436559	-2.45581277101531	-3.88883045835817
O	-0.53952893464730	-2.56959482384202	-4.66456300347049
O	-1.48254324114304	-2.82443334603063	-2.65757895446165
H	-6.21579307768333	-0.44250673421780	-5.20017740281080
H	-4.50702569130703	-0.66483439282530	-6.99239801501484
H	-2.25345500183177	-1.60501906817146	-6.41624022166634
C	-0.69782317140620	-4.95878261920835	0.64003266882464
H	-0.14246551396756	-4.26855314544318	0.00425929158428
C	-1.56499901061912	-5.76169723529325	-0.28263528750829
H	0.04097762168212	-5.62337417115301	1.10470835028194
C	-1.27008614975821	-7.10705443203633	-0.48477776389521
N	-2.55971880915938	-5.14675688352479	-0.93119675228049
C	-3.32233196053260	-5.88802200572453	-1.74701431418337
C	-2.03364083643072	-7.85185842814217	-1.36209221680887
C	-3.09570688188757	-7.23057545412482	-1.99390188691742
H	-0.44897027966081	-7.55568263001074	0.05970576704431
C	-4.49255483042976	-5.19699228917258	-2.40166217730900
H	-1.81591382668513	-8.89875323225661	-1.53565637118953
H	-3.75320828609734	-7.75722956558907	-2.67097355212769
O	-4.59272145865637	-3.95115450698746	-2.14850171935458
O	-5.26172024969600	-5.84458137779236	-3.10291674408869
Lu	-3.15924510547477	-2.57956966384080	-1.27188069373515
O	-3.48382066515627	-2.65056936457145	0.95153500352491
H	-4.32199051672265	-3.13099640579158	1.10808503700199
H	-2.74636878305919	-3.26726325002240	1.26552186617440

M = Pb^{2+}

O	-2.76668903978355	-0.00075975883610	-1.19136157060081
C	-2.03463458705198	0.54596944422670	-0.11275530248274
C	-0.75554437889742	-0.21743589965196	0.06360688548233
H	-2.62251864272665	0.51834898877597	0.81333392378930
H	-1.78576719763691	1.59316009523503	-0.32466292118776
O	-1.01820011054133	-1.49439153143203	0.61136950075269
H	-0.09280829144053	0.34450668075609	0.73373202332672
H	-0.25096329396511	-0.31980890980580	-0.90518520099746
C	0.14673363682362	-2.29808685325047	0.67391878263544
C	-0.06898623773483	-3.44219334949209	1.63211209124820
H	0.98612662622305	-1.70070437476641	1.05079477890164
H	0.41090111683702	-2.63618807415438	-0.33489593659411
N	-1.08433180616846	-4.41133447513022	1.22481709196533
H	-0.37511778452364	-3.01255303406080	2.58716500246283
H	0.90541876224596	-3.93007482503926	1.80151716484286
C	-1.58195442868364	-5.13107427658032	2.39442696210414
C	-2.97043419916213	-5.71555387153097	2.27190795658734

H	-1.60407972120882	-4.42716816220503	3.22826224545151
H	-0.90043537013911	-5.94685155505343	2.68770046625223
O	-3.90632291862361	-4.69331862068958	1.99546141692358
H	-3.21280414054919	-6.19685941826779	3.22921305130178
H	-3.03517849099005	-6.48508912083556	1.49660329110471
C	-6.89951292084176	-3.26232485436305	-0.65930172235506
C	-6.68672025819680	-1.76502015105536	-0.57817121466653
N	-5.55455126452581	-1.26528315577382	-1.37092703320308
H	-6.51046707477688	-1.49290080019522	0.46409271442973
H	-7.61640991534467	-1.25825716183651	-0.87952288572470
C	-5.18777655875215	0.05887172949969	-0.85509157636251
C	-3.96493538202128	0.70041144733146	-1.47158765933625
H	-5.02040161639507	-0.04442196439190	0.21957122735768
H	-6.03037446685349	0.75745047021196	-0.98672775314288
H	-3.89776109061733	1.72455043744154	-1.08975959031449
H	-4.05472392783875	0.77617406543691	-2.55636843332578
C	-5.92134143614419	-1.22094501637909	-2.78021871230991
O	-5.91692668137445	-4.00901406835844	0.03148322998786
H	-6.86665134391749	-3.60963141024530	-1.69326779815832
H	-7.89287269496482	-3.49807605980919	-0.25940634881948
C	-6.15856560119840	-4.13677712845037	1.41750996341487
C	-5.23409266626717	-5.17388724673260	1.98658918385583
H	-7.19185611204512	-4.46584757081597	1.58583390451480
H	-6.01994100703022	-3.18073210642781	1.93744403968344
H	-5.29419230069664	-6.08611834875621	1.37936187542438
H	-5.55306485410460	-5.42298419967137	3.00715012037009
C	-4.77941455614745	-1.23989989140164	-3.76177508033597
H	-6.55566438298434	-0.34846051686416	-2.99473846309286
H	-6.53086734194512	-2.10075613164419	-3.00310266351967
N	-3.64930441527085	-1.85361468771340	-3.42976447503668
C	-4.94354791851959	-0.66711381492348	-5.02072935014677
C	-3.91164338881859	-0.74503100471700	-5.93633097600367
C	-2.64346513811020	-1.92367405540119	-4.30242008924623
C	-2.73450424296499	-1.38119734843110	-5.57233001395038
C	-1.37461884605146	-2.62716797999073	-3.85076310119043
O	-0.43404829362378	-2.68352335412958	-4.64536736992459
O	-1.37885809568907	-3.10812853756375	-2.67509051038582
H	-5.87159584223976	-0.16518878382125	-5.26479753363267
H	-4.01914671916769	-0.30762715665203	-6.92168779898437
H	-1.89290908955077	-1.46209127682973	-6.24541157848302
C	-0.52655446699072	-5.29691054476843	0.21170703505764
H	-0.06712574214138	-4.67560146457175	-0.56159453701901
C	-1.50934162823722	-6.20501513748732	-0.47692521600859
H	0.27443974952710	-5.91964384686653	0.64125968818742
C	-1.31205732513732	-7.58193067455406	-0.46786960273786
N	-2.53197262998645	-5.65601943478359	-1.13354004588920
C	-3.40049374530552	-6.43873647411626	-1.77028263919372
C	-2.21113496779466	-8.40050521961330	-1.12954756659461
C	-3.28296971594370	-7.82173296996585	-1.78588171246201
H	-0.46564352987142	-7.99796663727372	0.06379163965739
C	-4.53394428919032	-5.76392302401180	-2.52427497009067
H	-2.07823836837128	-9.47579818010381	-1.12883883399013
H	-4.01863546618755	-8.41123453963291	-2.31436940062436
O	-4.48726649880861	-4.49799094635388	-2.62260357620805
O	-5.41076052124518	-6.48091971042195	-3.01151809855914
Pb	-3.26823150362220	-3.05266521710611	-1.22023265431050

O	-3.20585493728085	-1.94398373802233	2.50093867819567
H	-3.44659040553860	-2.86944845040113	2.34279721315739
H	-2.46319586518336	-1.77982655473050	1.90271656877647

M = Ra^{2+}

O	-2.56852003454788	-0.07098002207476	-1.27758152831354
C	-1.84517912343553	0.53263463091645	-0.22549836795453
C	-0.63351247443750	-0.29511374360129	0.08204405680009
H	-2.46601091855789	0.63892946081725	0.67265341150459
H	-1.50977297865880	1.53240761884299	-0.52660018416092
O	-1.01618894025585	-1.52593836959576	0.66687268019842
H	0.00729252127941	0.25983778549459	0.77764207699373
H	-0.06372558111602	-0.47900226364113	-0.83700728958934
C	0.10466279688669	-2.32595398996324	0.99303500330278
C	-0.31716684607093	-3.44057721030302	1.91224267614168
H	0.84654255295279	-1.71825673186784	1.52473699940340
H	0.58220141167667	-2.68793693561508	0.07487098015291
N	-1.23039076906975	-4.42660030701319	1.32937082544755
H	-0.81254717968086	-2.98495937520687	2.77116996593269
H	0.59441233462837	-3.92627550623332	2.29523762403393
C	-1.86984564198337	-5.17831999183662	2.41305795679412
C	-3.19094435530680	-5.82111828243695	2.08085569563178
H	-2.04618755998902	-4.48595030897136	3.23863556183264
H	-1.19750486677691	-5.96178453860666	2.79528469706590
O	-4.15541243679620	-4.82044457925797	1.80017667201728
H	-3.51589530675521	-6.40549231015413	2.95064267532294
H	-3.11774345773230	-6.50509710858148	1.23250663990761
C	-7.06835743318418	-2.42282284686997	0.02809425120084
C	-6.52931356135120	-1.03213020657864	-0.19021205650378
N	-5.47787744874965	-0.89305326344675	-1.19969250743709
H	-6.10845251289794	-0.69189463834891	0.75717694325197
H	-7.38596853646136	-0.37718342467075	-0.42070780643907
C	-4.94690906343389	0.46788218188309	-1.09523705704138
C	-3.62511185192131	0.72505942831122	-1.78278789351389
H	-4.82216960624066	0.68459677453992	-0.03220969103735
H	-5.67737759951374	1.19762795672987	-1.48262170135573
H	-3.38427688744560	1.78671363889228	-1.65900323437449
H	-3.68097665446573	0.53204185528785	-2.85251035376319
C	-6.01095005149138	-1.18262100438306	-2.52338281262367
O	-6.02491058189290	-3.30585057531354	0.38529737583084
H	-7.59843951307033	-2.81811635237271	-0.84618546641917
H	-7.79817456003036	-2.35475728734303	0.84233461785131
C	-6.44092075913792	-4.34607539158977	1.24336193866115
C	-5.36852094817449	-5.39034840478995	1.34054033065851
H	-7.34397199138324	-4.83118634381865	0.85364229229347
H	-6.67567031778761	-3.93235380906466	2.23221335170880
H	-5.20168240212142	-5.84904260613740	0.35929483599084
H	-5.70872110334316	-6.16775220493218	2.03496434959274
C	-4.99994642236081	-1.36950818924595	-3.61822068451684
H	-6.72660471748711	-0.40700614928625	-2.83683932936205
H	-6.57570842669785	-2.11646673005041	-2.45942086542416
N	-3.87246548934586	-2.01276370748920	-3.33993418127105
C	-5.29021859193933	-0.92170117185490	-4.89715077933728
C	-4.38755094984143	-1.15622486381598	-5.91241045662109
C	-2.98469373972700	-2.21700234321953	-4.31009110758193
C	-3.20990947534552	-1.81027655953535	-5.61328743483617

C	-1.67371671190768	-2.90504465394927	-3.94260606286220
O	-0.91508643883354	-3.19972540935489	-4.88265938025354
O	-1.46471063229370	-3.10770760216370	-2.72188282872007
H	-6.21634276223593	-0.39452215172528	-5.08728754560389
H	-4.59289520893602	-0.81985819023332	-6.92146781034856
H	-2.45891777260638	-2.00977622397041	-6.36367814155527
C	-0.46141974487638	-5.29545474842990	0.44566629525797
H	0.12144847378606	-4.65676652435880	-0.22412531062013
C	-1.22320681194433	-6.24041522617212	-0.43461730468997
H	0.26747015101560	-5.87956826851867	1.02824632531387
C	-0.71355315247490	-7.50867780349690	-0.66054408102558
N	-2.31982397495399	-5.80550945443052	-1.04431914020923
C	-2.94245096140298	-6.60656041789316	-1.90535234908868
C	-1.34452815694178	-8.33586170964855	-1.56605064106400
C	-2.47642284876217	-7.87464531405996	-2.20523588805691
H	0.17429263406791	-7.83450051893069	-0.13429251409956
C	-4.21321664676219	-6.08320334830724	-2.56224826579287
H	-0.95973792887268	-9.32832881088368	-1.76699126680538
H	-3.01122963848001	-8.47360313658233	-2.92805940492707
O	-4.68097580540844	-5.01654431085515	-2.09883542260968
O	-4.68491690528544	-6.76111259388742	-3.49255520770699
Ra	-3.29767620603129	-3.04796852016137	-0.71470496054573
O	-3.70224338004217	-1.86378172191975	2.11754427583113
H	-3.84923870633039	-2.77100169767687	2.41169763944646
H	-2.76950261289612	-1.68720462498615	2.27218549468938
